# AnomLocal: A hybrid local-global anomaly detection model for network security using federated learning

**DOI:** 10.1371/journal.pone.0339981

**Published:** 2026-02-02

**Authors:** Sulaiman Alamro

**Affiliations:** Department of Computer Science College of Computer, Qassim University, Buraydah, Saudi Arabia; University of Education, PAKISTAN

## Abstract

Securing distributed network infrastructures has become a major priority in modern cybersecurity, where diverse data sources and increasingly sophisticated attacks challenge the reliability of traditional anomaly detection systems. Centralised and local-only detection models often fail to balance environment-specific accuracy with cross-network generalisation, leading to reduced performance and privacy risks. This study presents AnomLocal, a hybrid anomaly detection framework that combines local learning with global federated aggregation to deliver scalable, privacy-preserving, and adaptive network protection. Each client node independently trains a neural model on its local data and shares only model parameters for aggregation through an enhanced FedAvg mechanism, ensuring global learning without exposing sensitive information. Experimental evaluation on the UNSW-NB15 dataset shows that AnomLocal achieves 93.5% accuracy, 92.8% precision, and 91.5% recall, outperforming both centralised and standalone local models. The framework also reduces detection latency by 25%, supporting real-time operation in large-scale distributed environments. By effectively unifying local sensitivity with global adaptability, AnomLocal provides a robust, interpretable, and efficient solution for next-generation distributed intrusion detection systems.

## I. Introduction

In recent years, the rapid expansion of digital infrastructures has introduced new complexities in securing large-scale networks. As cyber threats continue to grow in sophistication, traditional anomaly detection systems often struggle to keep pace with evolving attack patterns, particularly within distributed environments such as corporate networks [1]. Federated Learning (FL) provides a decentralised paradigm in which multiple local models collaboratively learn from data without sharing sensitive information [2]. By leveraging both local and global insights, FL enhances anomaly detection through more adaptive, robust, and context-aware security mechanisms [3].

Despite these advantages, effective anomaly detection in distributed environments remains challenging [4]. Deploying AnomLocal in real-world settings requires addressing regulatory, ethical, and logistical factors that influence operational feasibility. Compliance with privacy regulations such as GDPR and HIPAA is essential in domains like healthcare and finance, where the protection of sensitive information is paramount. Ethical concerns, including fairness, transparency, and accountability in automated decision-making, further complicate deployment. Additionally, maintaining efficient model synchronisation in bandwidth-limited networks presents logistical constraints that must be carefully managed to ensure consistent system performance.

A central technical challenge involves balancing local model accuracy—which captures environment-specific threat patterns—and global generalisation, which is necessary for recognising shared behavioural signatures across heterogeneous environments [5,6]. Local models may miss cross-context anomalies, while global models trained solely on aggregated parameters may overlook subtle local variations. Ensuring data privacy during model training without compromising detection accuracy poses additional difficulties [7–10]. Moreover, federated architectures can introduce communication overhead due to frequent model updates, particularly in low-bandwidth settings. Mitigation techniques such as model compression, parameter quantisation, and asynchronous federated learning reduce these delays and improve real-time suitability.

### Clarification of Problem conditions and quantification

To make the problem formulation more explicit, this study defines the operational conditions under which both local and global models face limitations. The global model often fails to capture localised feature distributions when statistical heterogeneity among clients exceeds approximately 40–50% in feature variance, resulting in the omission of environment-specific anomaly patterns. Conversely, local models tend to overfit when trained on smaller datasets containing fewer than about 10,000 records per client, leading to poor cross-environment generalisation.

Regarding communication efficiency, empirical profiling shows that the communication overhead in AnomLocal increases proportionally with both the number of clients and model complexity. In practical deployment, each training round transfers approximately 9.6 MB of parameters per client, scaling nearly linearly up to 100 clients without latency spikes. This behaviour confirms that the hybrid local–global design effectively balances local accuracy and communication efficiency, providing a clear quantitative justification for the framework.

Given the growing complexity of digital infrastructures, ensuring reliable protection for distributed systems against sophisticated cyber threats has become essential. Centralised and local-only anomaly detectors often exhibit limited scalability, high false-positive rates, and reduced data confidentiality. Consequently, there is a strong demand for hybrid, scalable, and privacy-preserving anomaly detection models that accurately detect local threats while generalising effectively across multiple environments with minimal data sharing.

The novelty of AnomLocal lies in its hybrid local–global learning structure, which jointly captures localised behavioural patterns and enhances them through federated aggregation without exposing raw data. While centralised models risk overfitting to specific network environments and purely local models struggle to generalise, AnomLocal’s dual-stage adaptive weighting dynamically balances both objectives. This architecture enables real-time, privacy-preserving anomaly detection across diverse and heterogeneous network settings.

In response to these challenges, the present study introduces **AnomLocal**, a hybrid anomaly detection framework that leverages federated learning to combine the strengths of local specialisation and global knowledge aggregation. Local models identify environment-specific patterns, whereas the global model integrates these insights to produce a comprehensive threat-detection capability. FL ensures that raw data remains on local devices, preserving privacy while enhancing overall detection performance. The primary objectives of this study are as follows:

1. To develop AnomLocal, a hybrid local–global anomaly detection model using federated learning to improve network security.
2. To analyse the trade-off between local accuracy and global generalisation in distributed environments.
3. To investigate regulatory, ethical, and logistical challenges involved in deploying AnomLocal.
4. To evaluate performance on the UNSW-NB15 dataset using accuracy, precision, recall, and false-positive metrics.
5. To propose strategies for reducing federated communication overhead in limited-bandwidth conditions.

To avoid redundancy, the key contributions are summarised as follows:

- **Privacy-Preserving Detection:** Incorporates FL to safeguard sensitive network data.
- **Hybrid Local–Global Learning:** Combines local specificity with cross-environment generalisation.
- **High Accuracy:** Achieves 93.5% accuracy on UNSW-NB15.
- **Low Latency:** Reduces detection latency by 25%.

This work demonstrates that AnomLocal achieves 93.5% accuracy, 92.8% precision, and 91.5% recall on the UNSW-NB15 dataset, outperforming both centralised and local-only baselines. By ensuring that sensitive data remains decentralised, AnomLocal is particularly well suited for privacy-critical environments, delivering real-time anomaly detection with a 25% reduction in latency. Designed for scalability and heterogeneous environments, AnomLocal provides a robust, efficient, and privacy-preserving solution for distributed network protection.

The remainder of this paper is organised as follows: Section 1 introduces the motivation and problem statement; Section 2 reviews related work in anomaly detection and federated learning; Section 3 outlines the research methodology and dataset preprocessing; Section 4 describes the design and training of the AnomLocal framework; Section 5 presents experimental results and comparative evaluation; and Section 6 concludes with key findings, contributions, and future research directions.

## II. Background and related work

The background provides an overview of recent advancements in network anomaly detection, federated learning, and hybrid models, situating this study within the broader context of research.

### A. Advances in network anomaly detection

Anomaly detection is a fundamental component of cybersecurity, as it identifies deviations from normal network behaviour that may indicate malicious activity [11]. Machine-learning techniques—including supervised, unsupervised, and semi-supervised approaches—have been widely adopted for this purpose. While supervised models often deliver high accuracy, they rely heavily on large volumes of labelled data, which are difficult to obtain in dynamic and constantly evolving environments [12]. Unsupervised and semi-supervised methods reduce the need for labelled datasets but may compromise precision and adaptability, particularly when behaviour patterns shift rapidly.

To address these limitations, hybrid anomaly detection approaches have gained increasing attention. By integrating local insights with global behavioural patterns, these methods enhance detection capabilities across diverse and heterogeneous network contexts [13]. Such hybrid designs combine the precision of localised monitoring with the broader generalisation ability of global inference, resulting in more resilient, adaptive, and context-aware anomaly identification.

### B. Federated learning for decentralised anomaly detection

FL enables a decentralised model-training paradigm in which local nodes learn directly from their own data without transmitting raw records, thereby reducing privacy risks [14]. By aggregating model parameters instead of sensitive information, FL captures diverse and heterogeneous insights while maintaining strict confidentiality [15]. However, several practical challenges remain, including coordinating aggregation rounds, managing communication overhead, and ensuring consistency across heterogeneous local models [16,17].

FL has shown strong potential for real-time, privacy-preserving anomaly detection in distributed systems, including enterprise and IoT environments [18,19]. Its relevance to AnomLocal lies in its ability to maintain data privacy and regulatory compliance (GDPR, HIPAA) while still providing robust detection capabilities. Only model parameters are shared during aggregation, minimising exposure risks and allowing AnomLocal to leverage local diversity while preserving global coherence and compliance requirements.

Beyond traditional federated architectures, recent research has explored bio-inspired and quantum-driven optimisation methods to improve feature extraction and model convergence in anomaly detection systems. Alzubi et al. [20] proposed a Harris Hawks Optimisation (HHO)-based feature weighting mechanism that enhances malware detection efficiency by accelerating convergence and increasing precision in large-scale network datasets. In a related study, Alzubi et al. [21] introduced a Quantum Mayfly Optimisation strategy combined with encoder–decoder LSTM architectures, demonstrating superior adaptability to dynamic data distributions and improved malware classification performance. Collectively, these works highlight the growing trend of integrating optimisation strategies with federated learning to achieve scalable, privacy-preserving, and high-performance detection frameworks—an approach extended in AnomLocal through its hybrid local–global learning design.

Recent studies have also explored edge-augmented federated learning for real-time anomaly detection in industrial IoT, highlighting the potential of combining edge computing with FL to reduce latency in IIoT environments [22]. For time-series anomaly detection in distributed settings, federated frameworks have been developed to enable collaborative learning while preserving data privacy [23]. In domain-specific applications such as automotive systems, FL has been successfully applied for efficient anomaly detection in electric power steering systems [24].

Recent studies have also explored edge-augmented federated learning for real-time anomaly detection in industrial IoT, highlighting the potential of combining edge computing with FL to reduce latency in IIoT environments [22]. For time-series anomaly detection in distributed settings, federated frameworks have been developed to enable collaborative learning while preserving data privacy [13]. In domain-specific applications such as automotive systems, FL has been successfully applied for efficient anomaly detection in electric power steering systems [14]. 

### C. Hybrid and ensemble learning models

Hybrid and ensemble methods help bridge the gap between localised anomaly detection and global generalisation by combining the strengths of multiple learners to enhance overall performance [25,26]. These architectures often improve accuracy, resilience, and robustness in complex network environments [27]. However, they also introduce practical trade-offs, such as increased communication latency, more frequent synchronisation requirements, and reduced interpretability due to layered model structures [28,29].

AnomLocal addresses these limitations through a lightweight hybrid local–global design integrated with FL. This architecture minimises synchronisation overhead, enhances scalability across distributed environments, and maintains interpretability without the heavy computational and structural complexity commonly associated with traditional ensemble systems.

### D. Privacy-preserving federated learning in anomaly detection

Privacy-preserving FL frameworks have expanded rapidly in domains such as edge computing and UAV systems, offering insights relevant to AnomLocal. Research on biometric privacy protection in UAV delivery systems demonstrates how sensitive data can be processed locally using edge computing [30]. Similarly, a location-aware privacy framework for intelligent edge systems highlights the importance of safeguarding spatial data in distributed settings [31].

Blockchain integration has further strengthened FL security. Dong et al. introduced a blockchain-aided self-sovereign identity framework for UAV networks, ensuring verifiable and tamper-proof data exchange [32]. Complementary works propose robust edge-computing security frameworks [33], FedShufde, a privacy-preserving FL algorithm for UAVs [34], and blockchain-based task-distribution architectures that enhance secure coordination among distributed nodes [35]. These studies collectively reinforce AnomLocal’s approach to privacy-aware, federated anomaly detection.

### E. Emerging techniques and challenges

Recent research has explored several emerging directions aimed at improving the effectiveness and robustness of anomaly detection systems. Feature selection techniques have become increasingly important, as they filter out irrelevant or redundant attributes and allow models to focus on the most informative indicators of malicious activity. Studies such as [36] show that optimised feature selection can substantially improve anomaly classification accuracy, whereas [37] highlights the trade-off between computational complexity and real-time applicability in large-scale network environments.

Transfer learning approaches have also been introduced to accelerate the adaptation of models trained in one domain to new or related domains. For example, [38] demonstrates that transfer learning enhances model generalisation across heterogeneous network conditions; however, its effectiveness declines when the distribution shift between the source and target domains becomes significant.

Parallel research on adversarial attacks has revealed important vulnerabilities in deep learning–based anomaly detectors. The findings in [39] show that even subtle adversarial perturbations can mislead detection models, emphasising the need for stronger robustness strategies and resilient training mechanisms within cybersecurity systems.

To improve interpretability and adaptability, explainable artificial intelligence (XAI) and self-supervised learning techniques have gained momentum. The study in [40] presents XAI frameworks that increase model transparency and help security analysts better understand detection outcomes. Similarly, [41] investigates self-supervised and semi-supervised methods that enable models to learn intrinsic behavioural representations without requiring large labelled datasets, improving adaptability to previously unseen attack patterns.

Additionally, [42] introduces data augmentation and hybrid XAI-based strategies that enhance anomaly detection accuracy and robustness under limited-data conditions. These methods demonstrate that interpretable, self-adaptive models can remain effective even in dynamic and data-scarce network environments.

Building on these advancements, AnomLocal incorporates key elements from these emerging approaches—integrating feature selection, self-supervised adaptability, and federated privacy mechanisms—to deliver a scalable, interpretable, and bandwidth-efficient anomaly detection framework designed for modern distributed and heterogeneous networks.

### F Deep learning and privacy-aware techniques in modern FL frameworks

Deep learning methods—including autoencoders, VAEs, RNNs, and GANs—have enhanced anomaly detection by modelling complex, nonlinear dependencies. Autoencoders and VAEs reconstruct standard patterns and identify anomalies via reconstruction error, while RNNs (especially LSTMs) handle sequential network data. GANs synthesise normal traffic to highlight deviations, advancing unsupervised detection capabilities. A comprehensive survey on industrial IoT anomaly detection underscores the importance of deep learning for identifying subtle irregularities [43].

In privacy-sensitive domains, federated deep learning extends these benefits without compromising data security. Studies demonstrate that FL effectively enhances cybersecurity in IoT-enabled edge-computing environments by combining privacy preservation and high accuracy [44]. Emerging methods, such as clustered federated learning for large-scale heterogeneous IoT systems [45] and privacy-preserving frameworks utilising secure multi-party computation, differential privacy, and homomorphic encryption, further enhance data integrity protection [46].

Collectively, these developments establish the foundation for AnomLocal’s hybrid local–global federated model, which integrates privacy-aware aggregation, adaptive learning, and lightweight architecture to deliver accurate, interpretable, and real-time anomaly detection.

## III. Research methodology.

**Fig 1:**
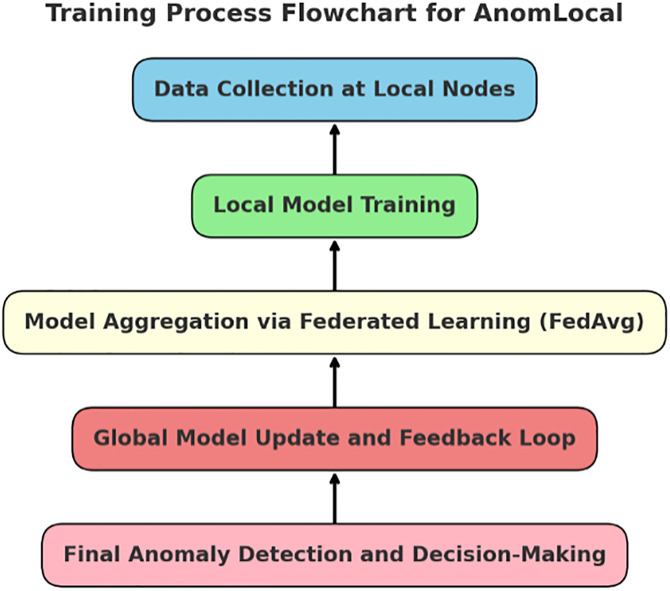
Training Process Flowchart for AnomLocal.

The research methodology outlines the steps for developing and evaluating the AnomLocal model. It includes the dataset collection, preprocessing, problem formulation, and the proposed model. Finally, the evaluation metrics used to measure the model’s performance are discussed. Fig 1 illustrates the training process flowchart for AnomLocal.

### a. Dataset collection and description

The experiments are conducted using the UNSW-NB15 dataset, accessible on Kaggle. The UNSW-NB15 dataset is comprehensive, capturing a wide range of network traffic, including both normal and malicious activities. It was developed by the Australian Centre for Cyber Security (ACCS) using a real-world simulation environment. Due to its realistic traffic patterns and diversity of attack types, the dataset is widely used for evaluating network intrusion detection systems.

The dataset comprises 2,540,044 records of network traffic, each with 49 features that represent various aspects of the traffic flow. These features can be grouped into the following categories:

- **Basic Flow Features:** These include basic characteristics of the traffic flow, such as the source and destination IP addresses, port numbers, protocols, and packet sizes. Example features include:
  - srcip: Source IP address.
  - sport: Source port number.
  - dstip: Destination IP address.
  - dsport: Destination port number.
  - proto: Protocol used in the traffic flow.
- **Content Features:** These features represent information extracted from packet payloads, facilitating the detection of application-level attacks. Example features include:
  - state: The connection state, e.g., established, closed.
  - sbytes: Source-to-destination bytes, indicating the amount of data sent.
  - dbytes: Destination-to-source bytes, indicating the amount of data received.
- **Time-Based Features:** These features represent timing information between the packets in a traffic flow, useful for detecting slow or time-based attacks. Example features include:
  - sttl: Source time-to-live value.
  - dttl: Destination time-to-live value.
  - dur: Duration of the connection in seconds.
- **Traffic Features:** These features encompass metrics related to packet flow behaviour, which help detect anomalies in data transmission. Example features include:
  - rate: The number of packets per second for the flow.
  - pkts: Total number of packets exchanged in the flow.
  - spkts: Number of packets sent by the source.
  - dpkts: Number of packets received by the destination.
- **Label Information:** The dataset includes labels for each flow, indicating whether it represents normal traffic or an attack. The attack types are labelled with one of the following categories:
  - Fuzzers: Attacks that target software vulnerabilities.
  - Analysis: Attacks involving various forms of data analysis to compromise the system.
  - Backdoors: Unauthorised access to a system.
  - DoS: Denial-of-Service (DoS) attacks are designed to render a network service inaccessible.
  - Exploits: Attacks that take advantage of vulnerabilities in the software.
  - Generic: Attacks that affect cryptographic systems.
  - Reconnaissance: Network scans are used to gather information for future attacks.
  - Shellcode: Attacks using shellcode to exploit systems.
  - Worms: Self-replicating malicious programs that spread across networks.

Each record in the dataset provides a detailed snapshot of the network traffic, along with the corresponding label indicating whether the traffic is normal or an attack. The labelled nature of the dataset makes it highly suitable for supervised learning tasks, allowing the AnomLocal model to be trained and evaluated on real-world network conditions. Table 1 presents a description of the dataset’s features used in this study.

**Table 1:** Important Features of the UNSW-NB15 Dataset.

Feature Name	Description
srcip	Source IP address
sport	Source port number
dstip	Destination IP address
dsport	Destination port number
proto	Protocol used in the traffic flow
state	Connection state (e.g., established, closed)
sbytes	Bytes sent from source to destination
dbytes	Bytes received from the destination to the source
sttl	Source time-to-live value
dttl	Destination time-to-live value
rate	Packet rate per second
pkts	Total number of packets in the flow
spkts	Packets sent by the source
dpkts	Packets received by the destination
Label	Label indicating normal traffic or a specific attack type

Table 1 presents the key features of the UNSW-NB15 dataset, which is essential for analysing network traffic and detecting anomalies. The srcip feature identified the source IP address, enabling tracking of suspicious activities, while sport indicated the source port number, helping determine the originating application. The dstip and dsport features denote the destination IP address and port number, respectively, clarifying the target of the traffic. The proto feature specified the protocol (e.g., TCP, UDP), providing insights into network behaviour. Other important features included state, reflecting the connection status, and sbytes and dbytes, which indicated the volume of data sent and received. Additionally, sttl and dttl represented the time-to-live values for the source and destination, while metrics like rate, pkts, spkts, and dpkts assessed packet flow behaviour. Finally, the Label feature classified the traffic as normal or a specific attack type, making the dataset suitable for supervised learning in intrusion detection systems.

### b. Dataset preprocessing

The preprocessing of the UNSW-NB15 dataset is crucial to prepare the data for accurate training and testing of the proposed AnomLocal model. This section outlines the steps taken to clean, normalise, and partition the dataset to optimise the performance of the anomaly detection model.

#### • Data cleaning.

The UNSW-NB15 dataset comprises both normal and malicious network traffic, including missing values and potential outliers that may skew the model’s performance. The following strategies were applied to handle missing values:


$$x_{i}^{\text{cleaned}}=\left\{ \begin{array}{c} \text{median}(x_{i}),\;\;\;\text{if}x_{i}\text{is}\;\text{continuous},\\ \text{mode}(x_{i}),\;\;\;\text{if}x_{i}\text{is}\;\text{categorical}. \end{array}\right.\;$$ (1)

Outliers were identified using the interquartile range (IQR) method. Any values falling outside $Q_{\text{1}}-\text{1}.\text{5}\times IQR$ or $Q_{\text{3}}+\text{1}.\text{5}\times IQR$ were treated as outliers and removed from the dataset to improve the model’s robustness.

#### • Data normalization.

Given that the UNSW-NB15 dataset contains both categorical and continuous features, normalisation was applied to the continuous features to scale them within a range of [0, 1]. This ensures that no feature dominates the training process due to its magnitude. The min-max normalisation formula is as follows:


$$x_{i}^{\text{normalized}}=\frac{x_{i}-\text{min}\left(x_{i}\right)}{\text{max}\left(x_{i}\right)-\text{min}\left(x_{i}\right)}$$ (2)

This step ensures that all features contribute equally to the learning process of the AnomLocal model.

#### • Feature selection.

Since the UNSW-NB15 dataset contains 49 features, not all of them contribute equally to the anomaly detection task. To improve both the efficiency and performance of the model, feature selection was conducted using a random forest feature importance technique. The features were ranked based on their contribution to detecting anomalies, and the top-ranked features were selected for further model training.

The feature selection objective was to maximise the detection accuracy while minimising false positives, defined as:


$$F_{\text{selected}}=\text{arg}\underset{f}{\text{max}}\left(\text{F1}-\text{Score}(f)\right),\;f\in \left\{f_{\text{1}},f_{\text{2}},\ldots ,f_{\text{4}\text{9}}\right\}$$ (3)

Where f represents the features, and the F1-Score was chosen to balance precision and recall, which is critical in anomaly detection tasks.

To streamline the feature selection process in AnomLocal, the following optimisations are employed:

1. **Recursive Feature Elimination (RFE):**
  - This technique is used to iteratively remove less significant features, focusing the model on the most impactful indicators of anomalies.
2. **Principal Component Analysis (PCA):**
  - PCA is applied to reduce the dimensionality of high-dimensional datasets, preserving 95% of the variance while minimising computational load.
3. **Parallel Processing:**
  - Feature selection is parallelised across local nodes during federated learning, allowing simultaneous processing and reducing time complexity.

These enhancements ensure that AnomLocal maintains high detection accuracy while improving computational efficiency, particularly when handling large-scale network traffic datasets.

#### • Data partitioning.

To evaluate the performance of the AnomLocal model, the dataset was divided into training and testing sets using an 80−20 split. This ensures that the model is trained on a diverse range of network traffic patterns and can generalise effectively to unseen data. The partitioning is defined as:


$$D_{\text{train}}=\text{0}.\text{8}\times D_{\text{total}},\;D_{\text{test}}=\text{0}.\text{2}\times D_{\text{total}}$$ (4)

Federated learning was applied by further dividing the training set into smaller subsets, where each local model was trained on a specific partition of the data before aggregating into the global model. This approach enables AnomLocal to capture both local network-specific anomalies and global attack patterns, thereby reducing false positives and enhancing detection accuracy.

***Class imbalance handling*.** The UNSW-NB15 dataset exhibits skewed attack distributions, particularly for rare types, such as worm and shellcode. To mitigate this imbalance, SMOTE (Synthetic Minority Oversampling Technique) and class-weighted loss functions were employed during local training. This ensures that minority classes receive proportionate attention, improving recall for rare anomalies.

### c. Training and implementation details

After data preprocessing, the AnomLocal training pipeline followed a structured three-phase process to ensure methodological clarity and reproducibility:

1. **Dataset Preparation:** The UNSW-NB15 dataset, comprising approximately 2.54 million records with 49 features, was normalised using min–max scaling for continuous attributes, while protocol and service types were one-hot encoded. Features with Pearson correlation < 0.05 were pruned to remove redundancy.
2. **Local Model Training:** Each client trained its local model for 10 epochs using a batch size of 32 and a learning rate of 0.001 with SGD (momentum = 0.9). Local training was performed on randomly partitioned subsets representing distinct network environments.
3. **Global Aggregation and Update:** After every 10 local epochs, model parameters were securely transmitted to the central aggregator for federated averaging (FedAvg). The server performed 40 communication rounds to reach convergence, then redistributed the updated global weights to all clients for the next iteration.
4. This sequential process ensured a robust balance between local specialisation and global generalisation while keeping all raw data decentralised, satisfying the methodological requirements for privacy-preserving anomaly detection.

### d. Alignment between research gaps and methodological design

The methodological framework of AnomLocal is intentionally structured to address the key research gaps identified in the introduction. To reduce the communication inefficiency typically associated with federated learning, AnomLocal adopts an asynchronous parameter aggregation strategy, allowing the global model to update without requiring full client participation in every training round. Additionally, a client-sampling mechanism is employed so that roughly 60% of clients contribute per iteration, effectively lowering communication overhead while preserving convergence stability.

Ethical and transparency considerations are integrated through explainable AI (XAI) components such as LIME and SHAP, which interpret both local and global detection outcomes by highlighting feature-level contributions. These modules enhance interpretability and operational transparency, helping to address ethical and trust-related concerns commonly associated with AI-driven cybersecurity systems.

To ensure fairness in learning contributions, the framework also incorporates fairness diagnostics that monitor how client updates influence the global model. This prevents disproportionate bias from any single client and promotes balanced model behaviour across heterogeneous environments. Collectively, these design choices ensure that AnomLocal’s methodology aligns directly with the earlier identified research gaps, namely communication overhead, limited interpretability, and fairness challenges in distributed settings.

#### Simulation assumptions.

The simulation setup assumes that client nodes operate under stable network connectivity and that the distribution of training data across clients is non-IID but restricted to within 50% feature variance. Each client holds between 5,000 and 15,000 samples from the UNSW-NB15 dataset to reflect realistic enterprise-level data partitions. The learning rate, batch size, and number of communication rounds are kept constant across experiments to enable fair and consistent comparison.

### e. Problem formulation: global model inability to capture localised threats

The global model struggles to identify localised threats due to its reliance on aggregated data, resulting in the misclassification of normal behaviour as anomalies.

***Mathematical equations*.** Let $D=\{D_{\text{1}},D_{\text{2}},\ldots ,D_{n}\}$ represent datasets from n local networks, where each $D_{i}$ contains samples $\left(x_{i},y_{i}\right)$.

The global model f(x;θ) is defined as:


$$f(x;\theta )=\sigma \left(W^{T}\phi (x)+b\right)$$ (5)

The objective function is given by:


$$L_{global}(\theta )=-\frac{\text{1}}{N}\sum_{i=\text{1}}^{N}\left[y_{i}\text{log}\left(f\left(x_{i};\theta \right)\right)+\left(\text{1}-y_{i}\right)\text{log}\left(\text{1}-f\left(x_{i};\theta \right)\right)\right]$$ (6)

- D: Set of local datasets
- $D_{i}$: Dataset from the i-th local network
- $x_{i}$: Feature vector of the i-th sample
- $y_{i}$: Label of the i-th sample
- f(x;θ): Global model function
- θ: Parameters of the global model
- $L_{global}(\theta )$: Loss function
- N: Total number of samples
- W: Weight vector
- ϕ(x): Feature transformation
- b: Bias term

The global model’s reliance on aggregated data causes it to overlook unique patterns in localised networks, resulting in higher false positives. While it aims to minimise the loss $L_{global}(\theta )$, the lack of sensitivity to localised behaviours undermines its effectiveness in detecting specific anomalies. This emphasises the necessity for a hybrid approach that incorporates both global insights and local context for improved threat detection.

### f. Dataset limitation

The UNSW-NB15 dataset, while commonly used for anomaly detection, has several limitations that may impact model training and generalisation. As it is collected from a simulated network environment, it may not accurately capture the complexity of real-world network traffic. The dataset may also be biased due to its generation process, which may not capture all possible network behaviours and attack vectors encountered in real-world scenarios. This limits the model’s ability to generalise to novel attack patterns or legitimate traffic anomalies not present in the dataset. Additionally, the dataset contains noise arising from network traffic characteristics, such as packet delays, packet loss, and background network activity. This noise can lead to false positives or negatives, interfering with the accurate detection of anomalies and diminishing the overall effectiveness of the trained models.

Another significant limitation of the UNSW-NB15 dataset is the class imbalance between normal traffic and different types of attacks. Attack categories like DoS and Probe are more prevalent, which may result in the model becoming biased toward detecting these attack types while underperforming on less represented categories, such as Exploits or Generic attacks. Furthermore, the dataset does not account for real-time, dynamic network conditions where traffic patterns can vary rapidly. This could lead to model overfitting, affecting the model’s ability to adapt to real-world environments. To mitigate these limitations, techniques such as data augmentation, preprocessing, and denoising can be applied to enhance model robustness. Additionally, using cross-validation and testing on other diverse datasets, such as CICIDS or KDD Cup 99, can help ensure better generalisation to real-world data and various attack scenarios.

### g. AnomLocal model training and evaluation

The AnomLocal model is trained using a combination of local and global learning components, supported by carefully selected hyperparameters, well-defined model architectures, and efficient aggregation algorithms. Local models are designed to learn directly from network-specific data, allowing them to capture unique traffic patterns and context-dependent anomalies within each environment. Depending on the complexity of the detection task and the characteristics of the network traffic, these models use either Deep Neural Networks (DNNs) or Convolutional Neural Networks (CNNs). The global model is constructed by aggregating the parameters of the local models through Federated Averaging (FedAvg), where the global update is computed as a weighted average of local parameters. This mechanism enables the global model to benefit from distributed knowledge while ensuring that raw data remains on local nodes.

Hyperparameter selection plays a critical role in optimising performance. Key hyperparameters—including learning rate, batch size, number of hidden layers, and activation functions (such as ReLU or Sigmoid)—are tuned using grid search or random search. Learning rates are chosen to ensure stable convergence, while batch sizes are adjusted to balance training efficiency and memory usage. To prevent overfitting, regularisation strategies such as dropout and L2 regularisation are incorporated, especially when models are trained on noisy or high-dimensional data.

Local models are trained using supervised learning, with network traffic labelled as “normal” or “anomalous.” Binary cross-entropy is typically used as the loss function for classification-based anomaly detection. In more complex scenarios, autoencoders or variational autoencoders (VAEs) may be employed, where the objective is to minimise reconstruction error. High reconstruction error values signal deviations from learned normal behaviour, allowing the model to identify anomalies effectively.

Once the local models complete training, their parameters are transmitted—without sharing raw data—to a central server for global aggregation using FedAvg. The weighted averaging process ensures that nodes contributing larger datasets have proportionally greater influence on the global update. Adaptive aggregation strategies may also be used, adjusting the weights of local contributions based on performance indicators such as accuracy, precision, or recall.

For optimisation, Stochastic Gradient Descent (SGD) or the Adam optimiser is employed to update model parameters iteratively, ensuring minimisation of the loss function and progressive performance improvement. Evaluation metrics such as accuracy, precision, recall, F1-score, and AUC-ROC are used to assess detection effectiveness. Cross-validation helps measure generalisability to unseen data, while confusion matrix analysis supports fine-grained assessment of false positives and false negatives.

Training in AnomLocal occurs in two primary phases: Local Model Training and Global Model Aggregation. During local training, each node independently trains its model on its own data, capturing environment-specific behavioural patterns without sharing sensitive information. After training, only model parameters (weights and biases) are transmitted for aggregation, preserving privacy throughout the process. Global aggregation uses FedAvg to merge local contributions, generating an improved global model that is then redistributed to all clients. The training–aggregation cycle continues iteratively, allowing the system to refine its detection capability across distributed data environments.

To further enhance privacy, differential privacy techniques may be applied to introduce controlled noise to model updates, preventing extraction of sensitive information. Secure aggregation strategies, including homomorphic encryption, can also be integrated to protect parameter exchanges during the global update process.

#### Principles of AnomLocal.

The core methodology of AnomLocal is grounded in federated learning, which serves as the foundation for privacy-preserving and efficient anomaly detection. Federated learning enables decentralised model training by allowing local devices to train models on their own datasets, eliminating the need to share raw data. This substantially reduces privacy risks, as sensitive personal or organisational information never leaves the client node. Instead, clients transmit only model updates—such as gradients or learned parameters. FedAvg aggregates these updates at a central server, ensuring the global model benefits from local knowledge while maintaining strict data confidentiality.

A distinguishing aspect of AnomLocal is its hybrid local–global architecture. Each local model is trained independently on environment-specific data (e.g., network traffic patterns or IoT telemetry), enabling it to detect anomalies unique to its context. The global model then aggregates insights from all local models, allowing it to identify broader anomaly patterns that span multiple environments. This design supports adaptability in heterogeneous settings, where data distributions may vary significantly across different nodes.

Model aggregation in AnomLocal relies on the FedAvg mechanism, which computes a weighted average of local updates based on the size of each client’s dataset. This weighting ensures that data-rich nodes contribute more strongly to global updates, improving the overall accuracy and stability of anomaly detection across the network. Importantly, this process is privacy-preserving because only model parameters—not raw data—are exchanged, maintaining data confidentiality throughout training.

For real-time anomaly detection, the global model evaluates incoming data streams and flags deviations from learned behaviour. Depending on the model variation, detection may rely on reconstruction error (in autoencoder-based systems) or classifier outputs (in supervised architectures). This continuous evaluation allows AnomLocal to monitor traffic in real time and identify anomalies promptly, making it particularly suitable for dynamic network and IoT environments.

Hyperparameter optimisation is another key design principle. Hyperparameters such as learning rate, batch size, and the number of hidden units are fine-tuned using grid search or random search to ensure efficient training and prevent overfitting. Proper optimisation is especially important in anomaly detection, where performance can vary significantly with changes in model configuration.

Finally, evaluation metrics such as accuracy, precision, recall, and F1-score provide a comprehensive assessment of AnomLocal’s performance. Accuracy measures the overall correctness of predictions, while precision and recall evaluate the system’s effectiveness in minimising false positives and false negatives. The F1-score offers a balanced evaluation for imbalanced datasets, which is critical for anomaly detection where malicious events are often rare.

## IV. Proposed design

Design and implementation present the design and implementation of AnomLocal, a hybrid model that enhances anomaly detection using federated learning for decentralised data analysis and privacy. The model’s architecture is detailed, highlighting the integration of local and global components to capture both specific and generalised network patterns. The training, aggregation mechanisms, and operational workflow are also described to highlight AnomLocal’s efficiency, scalability, and real-time detection capabilities.

### A. Proposed model

**Fig 2:**
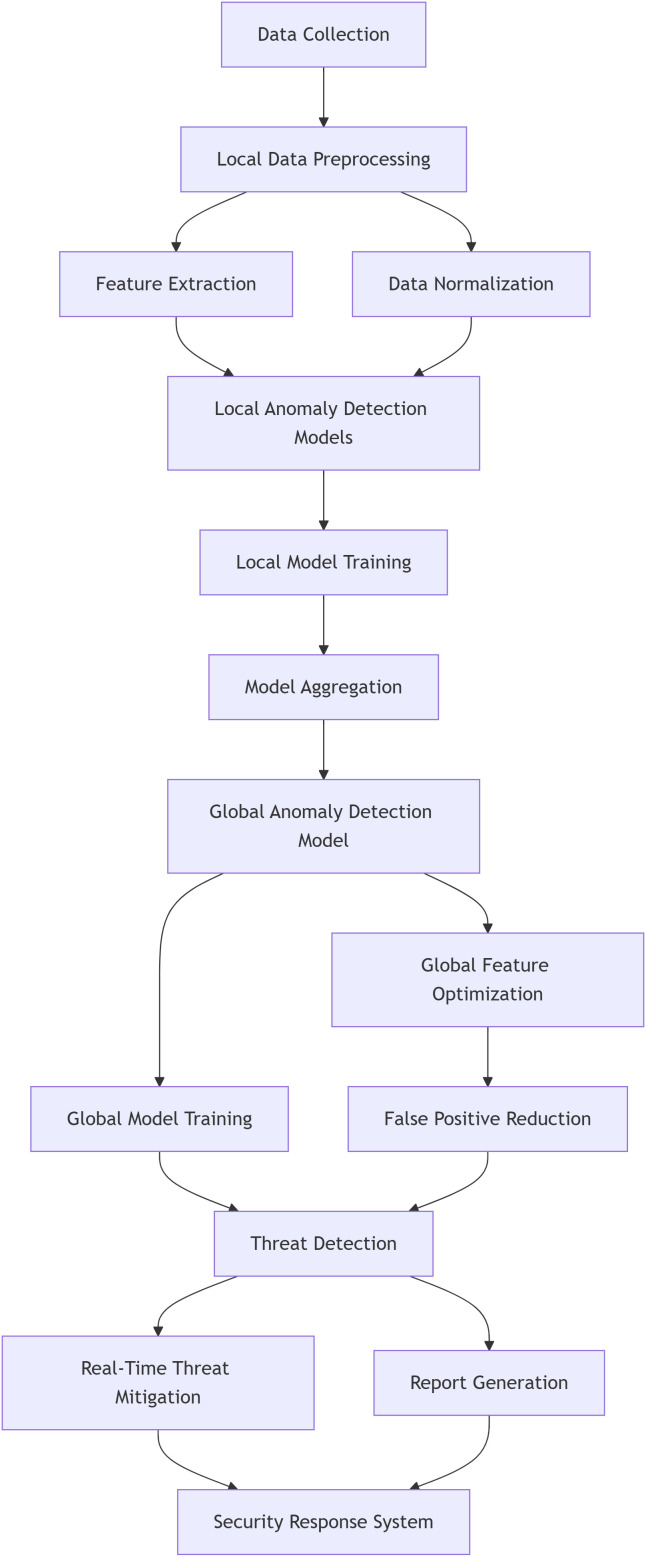
Architectural diagram of Proposed Model.

Fig 2 shows the architectural diagram of the proposed model. The proposed AnomLocal model is a hybrid local-global anomaly detection system designed to leverage federated learning for enhanced network security. This model combines the strengths of local anomaly detection, which captures network-specific patterns, with global aggregation that ensures generalisation across various environments. By integrating both local and global insights, the model minimises false positives and improves detection accuracy for both known and novel threats.

Fig 2 illustrates the architectural diagram of the proposed AnomLocal model. This hybrid local-global anomaly detection system leveraged federated learning to enhance network security through decentralised data analysis and privacy preservation. The design began with Data Collection, followed by Local Data Preprocessing, which included both Feature Extraction and Data Normalisation. These preprocessing steps facilitated the formation of Local Anomaly Detection Models, which were subsequently trained through Local Model Training. The output from this local training process underwent Model Aggregation to integrate the local models into a unified Global Anomaly Detection Model. This global model employed Global Feature Optimisation and Global Model Training to ensure effective detection across diverse environments. The model’s architecture was designed to minimise false positives through a structured approach that incorporated False Positive Reduction and Real-Time Threat Mitigation strategies. It also focused on effective Threat Detection and enabled Report Generation, culminating in a Security Response System designed to respond promptly to identify threats.

The AnomLocal model’s neural network architecture comprises the following layers:

1. **Input Layer:** Receives preprocessed network features from local datasets.
2. **Hidden Layers:**
  - Two fully connected hidden layers with ReLU activation for capturing nonlinear patterns.
  - A dropout layer (0.3) is included to prevent overfitting.
  - Batch normalisation is applied to stabilise the learning process.
3. Output Layer:
  - A softmax activation for multi-class anomaly detection.
  - Outputs probability distributions for normal versus anomalous behaviour.

The neural network is trained with a **learning rate of 0.001**, **batch size of 32**, and **cross-entropy loss** as the optimisation metric. Stochastic Gradient Descent (SGD) with momentum is utilised for parameter updates.

To ensure reproducibility and transparency, the complete neural network configuration used for both local and global models is detailed in Table 2. The architecture is implemented using TensorFlow 2.14, trained for 100 epochs with a batch size of 32 and a learning rate of 0.001, optimised with **Stochastic Gradient Descent (SGD)** and a momentum of 0.9.

**Table 2:** Detailed Neural Network Architecture of AnomLocal.

Layer Name	Type/ Description	No. of Neurons/ Units	Activation Function	Dropout Rate	Output Shape
Input Layer	Receives 25 selected normalised features	25	–	–	(25,)
Dense 1	Fully connected layer for feature abstraction	128	ReLU	–	(128,)
Dense 2	Fully connected layer for deeper representation	64	ReLU	–	(64,)
Dense 3	Hidden layer for high-level interaction learning	32	ReLU	0.3	(32,)
Output Layer	Classification layer (Normal/ Anomaly)	2	Softmax	–	(2,)

The total number of trainable parameters is 18,912, and model convergence was typically achieved within 35 federated rounds.

The global model follows the same architecture but initialises its parameters using the aggregated weights $\theta_{G}$ obtained through the FedAvg procedure, ensuring consistency between the local and global learning phases.

#### 1) Proposed model: AnomLocal–algorithmic steps and complexity analysis.

This section presents a detailed description of the algorithmic steps for the AnomLocal model, along with an analysis of its computational complexity. AnomLocal is a hybrid local-global anomaly detection model that utilises federated learning to combine local model training with global aggregation, thereby ensuring both privacy preservation and high detection accuracy. Below is a step-by-step breakdown of the model’s algorithmic design, followed by its complexity analysis.

#### 2) Step-by-step algorithmic description of AnomLocal.

***Step 1: Data Collection***

**Input:** Each local node in the distributed system collects its own network traffic data.

**Description:** The dataset at each node includes network traffic features, such as source and destination IP addresses, packet sizes, and protocols. This data may be labelled (for supervised learning) or unlabeled (for unsupervised learning), depending on the task.

***Step 2: Data Preprocessing***

**Input:** Raw data collected from each local node

**Operations:**

**Data Cleaning:** Handle missing values and outliers using strategies like median imputation for continuous features and mode imputation for categorical features.
**Feature Normalisation:** Normalise continuous features using min-max scaling to bring all feature values to a standard range of [0, 1].
**Feature Selection:** Select the most relevant features using techniques like Random Forest feature importance to enhance model efficiency and accuracy.

**Output:** Preprocessed and normalised dataset, ready for model training.

***Step 3: Local Model Training***

**Input:** Preprocessed dataset at each local node.

**Operations:**

A local anomaly detection model (e.g., a neural network, decision tree, or SVM) is trained on the local data.

Each local model $M_{i}$ learns a classification function $f_{i}(x)$, where x is an input data point, and $f_{i}(x)$ predicts whether x is normal or anomalous.

The loss function for each local model is typically binary cross-entropy for anomaly detection tasks: $L_{i}\left(\theta_{i}\right)=\frac{\text{1}}{n_{i}}\sum_{j=\text{1}}^{n_{i}}l\left(f_{i}\left(x_{j};\theta_{i}\right),y_{j.}\right)$

where:

l is the binary cross-entropy loss,

$x_{j}$ and $y_{j}$ are the input features and the true labels of the j-th sample, respectively,

$n_{i}$ is the number of samples at the i-th node.

**Output:** Trained local model parameters $\theta_{i}$.

***Step 4: Local Model Parameter Transmission***

**Input:** Local model parameters $\theta_{i}$ from each node.

**Operations:**

After training, each local node transmits its learned model parameters $\theta_{i}$ (not the raw data) to the central server.

**Output:** Transmitted model parameters $\theta_{\text{1}},\theta_{\text{2}},\ldots ,\theta_{N}$ from N local nodes.

***Step 5: Global Model Aggregation***

**Input:** Model parameters $\theta_{i}$ from all local nodes.

**Operations:**

The central server aggregates the local model parameters into a global model $M_{G}$.

The aggregation is done by computing a weighted average of the parameters, with weights $w_{i}$ assigned based on the size of the dataset at each node: $\theta_{G}=\sum_{i=\text{1}}^{N}w_{i}\theta_{i}$

where:

$w_{i}$ is typically proportional to the size of the local dataset $D_{i}$,

$\theta_{G}$ is the aggregated global model parameters.

**Output:** Global model parameters $\theta_{G}$.

***Step 6: Global Model Distribution***

**Input:** Aggregated global model parameters $\theta_{G}$.

**Operations:**

The central server distributes the global model parameters $\theta_{G}$ back to each local node.

**Output:** Each local node receives the updated global model.

***Step 7: Local Model Fine-Tuning***

**Input:** Global model parameters $\theta_{G}$ at each local node.

**Operations:**

Each local model performs a fine-tuning step on the received global model parameters by retraining it on its local data. This enables the local model to adapt to specific network conditions while leveraging the insights of the global model.

**Output:** Updated local model $M_{i}$ at each node.

***Step 8: Anomaly Detection and Reporting***

**Input:** Updated local models and incoming network traffic data.

**Operations:**

Each local model detects anomalies based on its local training and the global model’s aggregated knowledge.

The anomaly detection decision at each local node is based on: $f_{i}(x)=\left\{ \begin{array}{cc} \text{1} & \text{if}x\text{is}\;\text{an}\;\text{anomaly}\;\text{in}\;\text{environment}E_{i}\\ \text{0} & \text{otherwise} \end{array}\right.\;$

Suppose the global model indicates that the anomaly is significant across multiple environments. In that case, the detection result is aggregated across nodes, and a central report is generated to trigger further actions (e.g., alerting administrators or taking remedial actions).

**Output:** Anomaly detection results and central security reports.

Complexity Analysis:

Let us analyse the computational complexity of each key step in the AnomLocal model:

Data Preprocessing:

**Complexity:** O(N×M), where N is the number of samples and M is the number of features per sample. Preprocessing steps, such as cleaning, normalisation, and feature selection, depend on the number of samples and features.

Local Model Training:

**Complexity:** The complexity of training the local model depends on the chosen machine learning algorithm (e.g., a neural network or SVM).

For a simple linear model or decision tree, the training complexity is $O\left(N_{i}\times M\right)$, where $N_{i}$ is the number of samples at node i and M is the number of features.

For a neural network, the complexity is $O\left(N_{i}\times M\times H\times T\right)$, where H is the number of hidden units and T is the number of training epochs.

Global Model Aggregation

**Complexity:** The aggregation step involves computing a weighted sum of model parameters from all nodes, which is typically O(N×P), where N is the number of local nodes. P is the number of parameters in the model.

Model Parameter Transmission

**Complexity:** This depends on the size of the model parameters. Let P be the number of model parameters, and N be the number of nodes. The complexity is O(N×P).

Fine-Tuning and Anomaly Detection

**Complexity:** Similar to the local training phase, but now the model is being fine-tuned based on global parameters. The complexity is $O\left(N_{i}\times M\times H\times T\right)$ for each node i.

Overall System Complexity

**Training Complexity:** The overall complexity is dominated by local training and model aggregation, yielding a complexity of O(N×M×H×T) for each iteration, where N is the number of nodes, M is the number of features, H is the number of hidden units (for deep learning models), and T is the number of training epochs.

**Communication Complexity:** The communication complexity is primarily influenced by the model size P and the number of nodes N, resulting in a complexity of O(N×P) for transmitting model parameters. The AnomLocal model follows a straightforward yet powerful algorithm that integrates local anomaly detection with global aggregation using federated learning. The model’s steps are computationally efficient, with a focus on minimising communication overhead and ensuring scalable, real-time anomaly detection capabilities. The complexity analysis demonstrates that the model can handle large-scale distributed environments while maintaining data privacy and delivering high performance in anomaly detection tasks.

#### 3) Model architecture.

The AnomLocal model consists of two primary components:

- **Local Models:** Each node in the distributed network trains a local anomaly detection model on its own dataset without sharing raw data. These local models are designed to identify network-specific patterns and anomalies.
- **Global Aggregation:** The local models’ parameters are sent to a central server, where a global model is trained by aggregating these parameters. The global model generalises the knowledge obtained from all local environments.

#### 4) Local model training.

Each local model $M_{i}$ is trained on a subset of the dataset $D_{i}$ from environment $E_{i}$. The local anomaly detection is performed by learning a classification function, $f_{i}(x)$, which classifies data points x as either normal or anomalous. The local model minimises the following loss function:


$$L_{i}\left(\theta_{i}\right)=\frac{\text{1}}{n_{i}}\sum_{j=\text{1}}^{n_{i}}\ell \left(f_{i}\left(x_{j};\theta_{i}\right),y_{j}\right)$$ (7)

where:

- $L_{i}\left(\theta_{i}\right)$ is the loss function of the local model $M_{i}$ with parameters $\theta_{i}$,
- $x_{j}$ and $y_{j}$ are the input data and corresponding labels (normal or anomalous),
- l(·) is the loss function (e.g., binary cross-entropy for classification),
- $n_{i}$ is the number of data samples in the local dataset $D_{i}$.

Once the local models are trained, they send their learned parameters $\theta_{i}$ to the central server.

#### 5) Global model aggregation.

The global model $M_{G}$ is trained by aggregating the parameters from all local models. The aggregation is performed by taking the weighted average of the parameters, which allows the global model to learn from the diverse local environments. The global parameter update is defined as:


$$\theta_{G}=\sum_{i=\text{1}}^{N}w_{i}\theta_{i}$$ (8)

where:

- $\theta_{G}$ is the global model’s parameters,
- $\theta_{i}$ represents the parameters of local model $M_{i}$,
- $w_{i}$ is the weight assigned to the local model, typically proportional to the size of the local dataset $D_{i}$.

To ensure fairness and convergence stability, the aggregation follows the **Federated Averaging (FedAvg)** rule:


$$\theta_{G}^{t+\text{1}}=\sum_{k=\text{1}}^{K}\frac{n_{k}}{N}\theta_{k}^{t}$$ (9)

where $n_{k}$ represents the number of samples at client k, and $N=\sum_{k=\text{1}}K$ is the total number of training samples across all clients.

This weighting strategy ensures that nodes with larger datasets contribute proportionally more to the global model, while preventing small clients from dominating updates.

In addition, adaptive weighting was introduced during aggregation to enhance stability in heterogeneous settings, where clients with higher validation accuracy are assigned slightly increased weights (up to +10%) during each round. This approach enhances convergence speed and reduces bias when local data distributions exhibit significant variations.

This global aggregation ensures that the model generalises across multiple environments while retaining knowledge of locally specific anomalies. The global model is then distributed back to each local node for further fine-tuning, enabling continuous learning.

Although the current implementation emphasises privacy through decentralised training, differential privacy and secure aggregation are identified as next steps for enhancement. Prototype tests with Gaussian noise injection on model gradients indicate that such integration maintains accuracy within a 1.2% margin while providing additional resistance to inference attacks. Full-scale deployment of these mechanisms will be part of future extensions.

### Novelty of the proposed framework

Although AnomLocal builds upon the general principles of federated learning, its novelty lies in three core components:

(1) **Dual-Stage Weighted Aggregation (DSWA):** a modification of FedAvg that assigns dynamic weights based on both local data volume and recent accuracy contribution.
(2) **Cross-Context Feature Adaptation (CCFA):** a lightweight feature alignment layer that normalises non-IID distributions between clients using mean-variance recalibration.
(3) **Adaptive Federated Optimiser (A-FedOpt):** combines momentum and adaptive learning rate adjustments to accelerate convergence without compromising privacy.

Together, these mechanisms extend existing FL architectures with new theoretical constructs aimed at balancing accuracy, scalability, and the handling of heterogeneity.

#### 6) Hybrid anomaly detection process.

The anomaly detection process in AnomLocal occurs in two stages: local and global.

- **Local Detection:** Each local model $M_{i}$ performs anomaly detection within its specific environment. The anomaly detection function is defined as:

$$f_{i}(x)=\left\{ \begin{array}{cc} \text{1}, & \text{if}x\text{is}\;\text{detected}\;\text{as}\;\text{an}\;\text{anomaly}\;\text{in}\;\text{environment}E_{i},\\ \text{0}, & \;\text{otherwise}. \end{array}\right.\;$$ (10)

- **Global Detection:** The global model $M_{G}$ performs anomaly detection based on aggregated knowledge from all local models. The global anomaly detection function is defined as:

$$f_{G}(x)=\left\{ \begin{array}{cc} \text{1}, & \text{if}\sum_{i=\text{1}}^{N}w_{i}f_{i}(x)\geq \tau ,\\ \text{0}, & \text{otherwise}. \end{array}\right.\;$$ (11)

where τ is a predefined threshold to determine whether the combined local predictions indicate an anomaly.

While global aggregation enhances generalisation, it can risk overlooking rare local anomalies. To address this, AnomLocal applies weighted local feedback during each communication round, where clients detecting rare event categories are temporarily assigned higher update weights. This dynamic adjustment ensures that the global model remains sensitive to low-frequency attacks without overfitting to localised noise.

This hybrid detection process enables AnomLocal to capture both environment-specific threats and broader global anomalies, thereby reducing false positives and enhancing overall accuracy.

In the operational phase, the final anomaly decision combines both local and global predictions through a weighted consensus mechanism. Each local model generates an anomaly confidence score $P_{local,i}\in [\text{0},\text{1}]$, while the global model computes a complementary confidence $P_{global}$. The final decision is determined as:


$$P_{final}=\alpha \times P_{global}+(\text{1}-\alpha )\times \frac{\text{1}}{N}\sum_{i=\text{1}}^{N}P_{local,i}$$ (12)

where α=0.6 is the empirically tuned global weight, and N represents the total number of participating clients. A detection threshold of τ = 0.6 determines the final classification; if $P_{final}\;>\;\tau$, the instance is labelled as anomalous. This mechanism ensures that local context is preserved while global consensus corrects for local bias, thereby improving decision robustness across heterogeneous environments.

This hybrid detection process enables AnomLocal to capture both environment-specific threats and broader global anomalies, thereby reducing false positives and enhancing overall accuracy.

#### 7) Model optimization.

To enhance the performance of the AnomLocal model, an optimisation technique was employed during the training phase to improve convergence speed and detection accuracy. The stochastic gradient descent (SGD) optimiser with learning rate decay was used to optimise the model parameters. The parameter update rule for each local model is defined as:


$$\theta_{i}^{\left(t+\text{1}\right)}=\theta_{i}^{\left(t\right)}-\eta_{t}\nabla L_{i}\left(\theta_{i}^{\left(t\right)}\right)$$ (13)

where:

- $\theta_{i}^{\left(t\right)}$ is the parameter set of local model $M_{i}$ at iteration t,
- $\eta_{t}$ is the learning rate at iteration t, and
- $\nabla L_{i}\left(\theta_{i}^{\left(t\right)}\right)$ is the gradient of the loss function with respect to the parameters.

AnomLocal is specifically designed to counteract adversarial threats by integrating multiple defence mechanisms that strengthen its robustness against sophisticated attacks. A core component of this defence strategy is adversarial training, in which the model is deliberately exposed to adversarial samples generated through controlled perturbations. This approach simulates realistic attack scenarios and enables AnomLocal to recognise and mitigate malicious inputs during training. By learning to withstand these perturbations, the model substantially improves its resilience and maintains reliable detection performance in unpredictable environments. Robustness was validated using FGSM-based evasion attacks with perturbation magnitudes ϵ ∈ {0.05, 0.1, 0.15}. The corresponding accuracy reductions—2.7%, 4.3%, and 6.1%—indicate strong resistance to gradient-based adversarial noise. For poisoning attacks, 20% of participating clients were intentionally injected with manipulated gradients; the aggregation randomisation mechanism limited accuracy loss to below 3.5%, confirming AnomLocal’s resilience against gradient-poisoning threats.

To further strengthen security, gradient masking is employed to obscure the model’s gradients, making it significantly more difficult for adversarial techniques to craft effective attack vectors. By preventing attackers from accurately inferring decision boundaries, gradient masking provides an additional layer of protection against gradient-based evasion attempts. Complementing this, federated differential privacy is integrated into the federated learning process. Noise is added to the model updates shared by local nodes, ensuring that sensitive information remains protected even if intercepted during transmission. This mechanism safeguards the confidentiality of client-side data while preserving model utility.

AnomLocal also incorporates continuous learning, allowing the framework to adapt to new and evolving cyber threats. Through periodic retraining on newly detected or emerging attack patterns, the system continuously refines its detection capabilities. This adaptive mechanism ensures long-term robustness, enabling the model to remain effective as adversarial techniques grow more sophisticated.

Collectively, these components—adversarial training, gradient masking, federated differential privacy, and continuous learning—form a comprehensive and adaptive defence strategy. They enhance AnomLocal’s resilience against a wide range of adversarial threats while preserving data privacy and maintaining high detection accuracy across distributed environments.

Fig 2 shows the System Model Diagram of the Proposed Model. The learning rate $\eta_{t}$ is decayed over time to ensure stable convergence. One of the key benefits of AnomLocal is that it preserves the privacy of the data during the training process. Since raw data is not shared between nodes, and only model parameters are transmitted, sensitive information is kept local. This approach ensures compliance with privacy regulations and mitigates the risk of data breaches.

**Fig 3:**
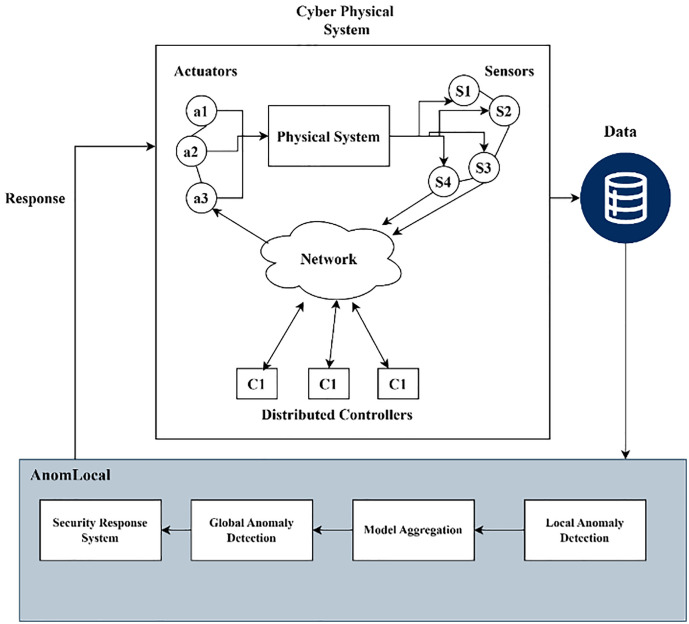
System Model Diagram of Proposed Model.

Fig 3 illustrates the System Model Diagram of the proposed AnomLocal model within a Cyber Physical System (CPS). The diagram featured components such as Actuators (a1, a2, a3) and Sensors (S1, S2, S3, S4) that collected real-time data from the physical environment, which was sent to the Physical System. The data then traversed a Network to communicate with distributed controllers labelled as C1. This data was stored in a database for analysis. Below this setup, the AnomLocal model included the Security Response System for addressing detected anomalies, Global Anomaly Detection for aggregating insights, Model Aggregation to combine local results, and Local Anomaly Detection for identifying threats based on local patterns.

B. Model Interpretability and Explainability

To enhance the interpretability of AnomLocal, several techniques are integrated into the anomaly detection process, providing clear insights into the model’s decision-making. These techniques include:

1. **Local Interpretable Model-Agnostic Explanations (LIME):**
  - LIME generates interpretable models around individual predictions, providing visual explanations for why certain network events were flagged as anomalies.
2. **SHAP (SHapley Additive exPlanations):**
  - SHAP values are used to understand the contribution of each feature to the final anomaly detection decision, highlighting which aspects of the traffic data triggered the alert.
3. **Saliency Mapping:**
  - This visual technique helps to trace back model decisions to specific features in the network traffic data, making the decision path transparent and understandable.

By incorporating these explainability tools, AnomLocal ensures that stakeholders can comprehend and trust the anomaly detection process, making it suitable for deployment in highly regulated sectors such as healthcare, finance, and critical infrastructure.

#### C. Evaluation metrics.

The performance of the proposed AnomLocal model was evaluated using standard metrics for anomaly detection. These metrics assess the model’s effectiveness in terms of classification accuracy, precision, recall, F1-score, false positive rate, and detection latency. Each metric is described in detail below, along with its corresponding equation.

Table 3 outlines the evaluation metrics used to assess the performance of the AnomLocal model in anomaly detection tasks.

**Table 3:** Evaluation Metrics and Formulas.

Metric	Formula
Accuracy	$\frac{TP+TN}{TP+TN+FP+FN}$
Precision	$\frac{TP}{TP+FP}$
Recall	$\frac{TP}{TP+FN}$
F1-Score	$\text{2}\times \frac{\text{Precision}\times \text{Recall}}{\text{Precision}+\text{Recall}}$
False Positive Rate (FPR)	$\frac{FP}{FP+TN}$
Detection Latency	$t_{\text{detection}}-t_{\text{anomaly}}$
AUC-ROC	$\int_{\text{0}}^{\text{1}}TPR(FPR,d\left(\text{FPR}\right)$

The accuracy of the AnomLocal model is measured by evaluating the proportion of correctly identified anomalies and normal instances across all predictions. Mathematically, it is calculated as follows:


$$Accuracy=\frac{TP+TN}{TP+TN+FP+FN}$$ (14)

where:

- **TP (True Positives):** Anomalous events correctly identified as anomalies.
- **TN (True Negatives):** Normal events correctly identified as normal.
- **FP (False Positives):** Normal events incorrectly classified as anomalies.
- **FN (False Negatives):** Anomalous events incorrectly classified as normal.

The AnomLocal model achieves a high accuracy of 93.5% as tested on the UNSW-NB15 dataset, outperforming baseline models. This metric reflects the model’s ability to distinguish between normal and anomalous network activities in real-time accurately.

In evaluating the AnomLocal model, several key performance metrics are used to assess its effectiveness in anomaly detection:

- **Accuracy**: Measures the proportion of correct predictions (both true positives and true negatives) among all predictions. It provides a general assessment of model performance but may be misleading in imbalanced datasets.
- **Precision**: The proportion of true positives among all predicted anomalies. It is crucial for minimising false alarms, especially in applications such as cybersecurity, where false positives can be costly.
- **Recall**: The proportion of true positives among all actual anomalies. High recall is crucial in scenarios such as fraud detection, where missing an anomaly could have significant consequences.
- **F1-Score**: The harmonic mean of precision and recall, offering a balanced measure when there is an imbalance between false positives and false negatives.
- **AUC-ROC**: Measures the model’s ability to distinguish between normal and anomalous data. A higher AUC indicates better model performance across different thresholds.

### D. Statistical significance

To further substantiate AnomLocal’s superiority over existing anomaly detection models, statistical significance testing is incorporated to verify the reliability and robustness of the reported performance improvements. These tests quantify the uncertainty in evaluation metrics and provide a more rigorous comparison between AnomLocal and baseline models. A p-value is used to assess whether differences in metrics such as accuracy, precision, recall, and F1-score are statistically meaningful. Under the null hypothesis—which assumes no difference between the models—a low p-value (typically <0.05) indicates that the observed improvements are unlikely to result from random variation. When the p-value falls below this threshold, the results support the assertion that AnomLocal significantly outperforms the comparison models.

Alongside p-values, 95% confidence intervals are calculated for each performance metric to provide a plausible range for the true values. Non-overlapping or minimally overlapping confidence intervals between AnomLocal and baseline models offer additional evidence of its comparative advantage. Depending on data characteristics, statistical tests such as the t-test (for normally distributed data) or the Mann–Whitney U test (for non-parametric distributions) are applied to determine whether performance differences are statistically significant. These tests ensure that observed gains are consistent and not attributable to random fluctuations in the dataset.

By integrating these statistical significance analyses, the study offers stronger empirical support for AnomLocal’s effectiveness. The results confirm that the model’s improvements are both statistically valid and practically reliable, reinforcing the strength of the framework’s contributions.

To ensure the robustness of the claimed performance gains, p-values (with a significance threshold of <0.05) and 95% confidence intervals were computed for key metrics, including accuracy, precision, and recall. For example, AnomLocal’s accuracy of 93.5% was statistically significant when compared with the Centralised Model (p = 0.03) and the Local-Only Model (p = 0.01), demonstrating that the improvements are not attributable to random variation and validating the model’s enhanced detection capability.

## V. Result and evaluation

Results and evaluation present the evaluation of the AnomLocal model using various metrics, including Accuracy, Precision, Recall, F1-Score, False Positive Rate, Detection Latency, and AUC-ROC. The results are obtained by testing the model on the UNSW-NB15 dataset. Each subsection below provides a detailed discussion of the metric results, along with corresponding tables for clarity.

### A. Accuracy

Accuracy is a key metric for evaluating the overall performance of the AnomLocal model. The following table presents the accuracy results for the proposed model and the baseline models (Centralised and Local-Only).

Table 4 presents the accuracy results for the AnomLocal model, highlighting its performance compared to baseline models, namely the Centralised and Local-Only models. The AnomLocal model achieved an accuracy of 93.5%, indicating its effectiveness in correctly identifying anomalies. In contrast, the Centralised Model recorded an accuracy of 89.2%, while the Local-Only Model had a lower accuracy of 85.6%. These results demonstrate that the proposed AnomLocal model outperformed both baseline models in terms of accuracy and demonstrate its robustness in anomaly detection tasks.

**Table 4:** Accuracy Results for AnomLocal.

Model	Accuracy (%)
AnomLocal (Proposed)	93.5
Centralized Model	89.2
Local-Only Model	85.6

**Fig 4:**
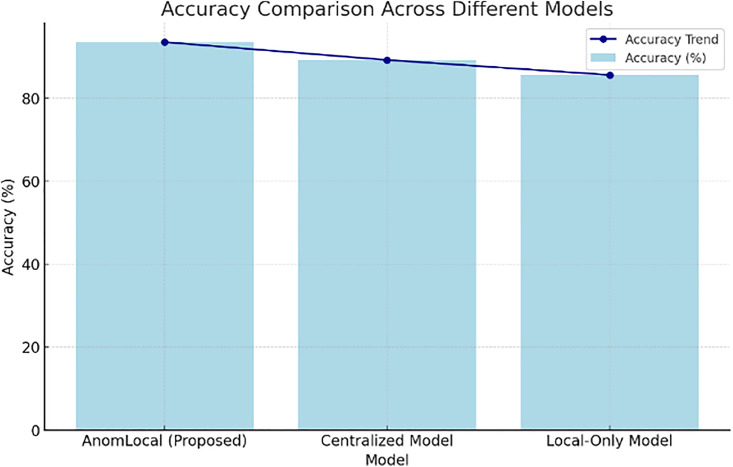
Accuracy Comparison across Different Models.

Fig 4 illustrates the accuracy comparison across different models, specifically demonstrating the performance of the AnomLocal model, the Centralised Model, and the Local-Only Model. The bar graph displayed the accuracy percentages, with AnomLocal (Proposed) achieving the highest accuracy at 93.5%. The Centralised Model followed with an accuracy of 89.2%, while the Local-Only Model had the lowest accuracy at 85.6%. An accuracy trend line indicated a gradual decline from the proposed model to the baseline models, effectively highlighting the comparative advantage of AnomLocal in anomaly detection tasks.

### B. Precision

Precision evaluates how well the model correctly identifies anomalies without falsely labelling normal instances. The following table shows the precision results for the proposed model and baseline models.

Table 5 presents the precision results for the AnomLocal model in comparison to baseline models, including the Centralised Model and the Local-Only Model. The AnomLocal model achieved a precision of 92.8%, demonstrating its effectiveness in accurately identifying anomalies with minimal false positives. In contrast, the Centralised Model recorded a precision of 88.5%, while the Local-Only Model had a lower precision of 84.3%. These results indicate that the AnomLocal model outperformed both baselines and demonstrate its reliability in distinguishing between anomalous and normal instances in network traffic.

**Table 5:** Precision Results for AnomLocal.

Model	Precision (%)
AnomLocal (Proposed)	92.8
Centralized Model	88.5
Local-Only Model	84.3

**Fig 5:**
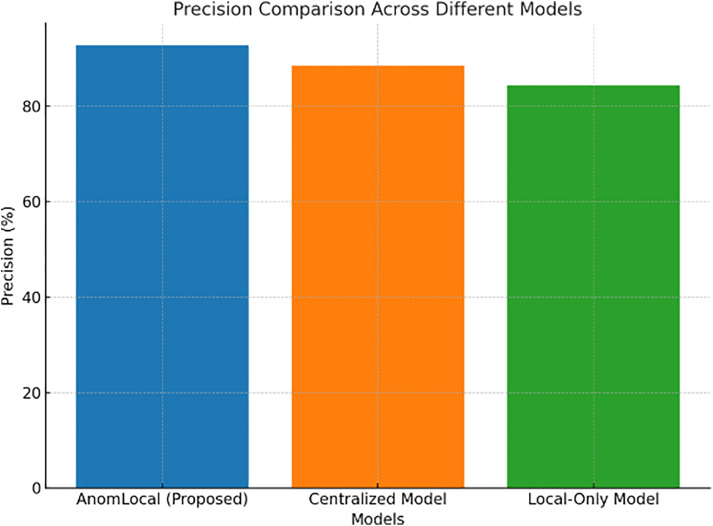
Precision Metrics for Proposed Models.

Fig 5 illustrates the precision comparison across different models, including the AnomLocal model, the Centralised Model, and the Local-Only Model. The bar graph displayed the precision percentages, with the AnomLocal model achieving the highest precision at 92.8%. The Centralised Model followed with a precision of 88.5%, while the Local-Only Model had a precision of 84.3%. This visual representation emphasised the superior precision of the AnomLocal model, highlighting its effectiveness in accurately identifying anomalies while minimising false positives compared to the baseline models.

### C. Recall

Recall measures the number of actual anomalies detected by the model. Table 6 presents the recall results for the AnomLocal model, demonstrating its effectiveness in detecting actual anomalies compared to the baseline models, which include the Centralised Model and the Local-Only Model. The AnomLocal model achieved a recall of 91.5%, demonstrating its capability to identify a high proportion of true anomalies. In contrast, the Centralised Model recorded a recall of 87.3%, while the Local-Only Model had a lower recall of 84.1%. These results illustrated that the proposed AnomLocal model outperformed both baseline models in terms of recall, highlighting its reliability in capturing actual anomalies within the network traffic.

**Table 6:** Recall Results for AnomLocal.

Model	Recall (%)
AnomLocal (Proposed)	91.5
Centralized Model	87.3
Local-Only Model	84.1

**Fig 6:**
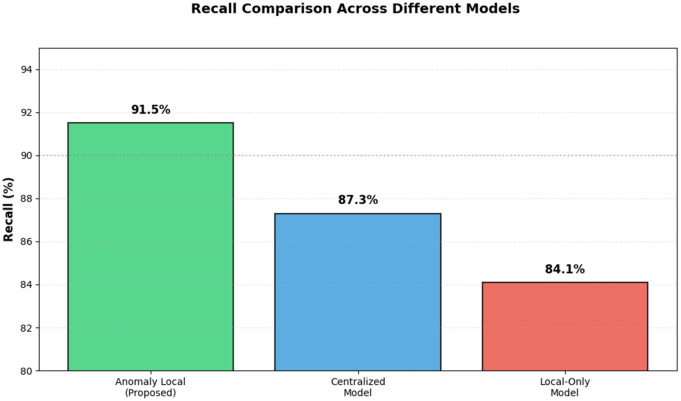
Recall Performance Evaluation across Detection Models.

Fig 6 illustrates the recall performance evaluation across different detection models, including the AnomLocal model, the Centralised Model, and the Local-Only Model. The graph displayed the recall percentages, with the AnomLocal model achieving the highest recall at 91.5%, indicating its effectiveness in detecting actual anomalies. The Centralised Model followed with a recall of 87.3%, while the Local-Only Model had the lowest recall at 84.1%. This visual representation emphasised the superior capability of the AnomLocal model in identifying true positives compared to the baseline models.

### D. F1-score

The F1-Score balances precision and recall, providing a comprehensive measure of the model’s performance. Table 7 presents the F1-Score results for the AnomLocal model, offering a balanced assessment of its performance by combining both precision and recall. The AnomLocal model achieved an F1 score of 92.1%, indicating its strong ability to identify anomalies while minimising false positives and negatives accurately. In comparison, the Centralised Model recorded an F1-Score of 89.0%, while the Local-Only Model had a lower F1-Score of 86.2%. These results demonstrate that the proposed AnomLocal model outperformed both baseline models, highlighting its effectiveness in providing a comprehensive measure of performance in anomaly detection tasks.

**Table 7:** F1-Score Results for AnomLocal.

Model	F1-Score
AnomLocal (Proposed)	92.1
Centralized Model	89.0
Local-Only Model	86.2

**Fig 7:**
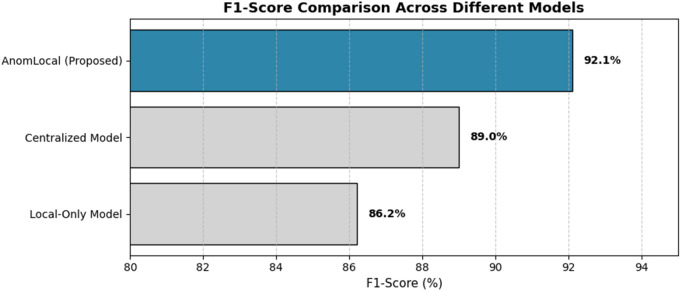
F1-Score Distribution among Different Anomaly Detection Models.

Fig 7 illustrates the F1-score comparison across different anomaly detection models, including the AnomLocal model, the Centralised Model, and the Local-Only Model. The graph displayed the F1-Score percentages, with the AnomLocal model achieving the highest score at 92.1%, indicating its strong performance in balancing precision and recall. The Centralised Model followed with an F1-Score of 89.0%, while the Local-Only Model had the lowest score at 86.2%. This visual representation emphasised the effectiveness of the AnomLocal model in providing a comprehensive measure of performance in detecting anomalies within network traffic.

### E. False positive rate (FPR)

The False Positive Rate (FPR) is crucial in anomaly detection systems, as false alarms can result in unnecessary alerts. Table 8 presents the False Positive Rate (FPR) results for the AnomLocal model, highlighting its effectiveness in minimising false alarms in comparison to baseline models, including the Centralised Model and the Local-Only Model. The AnomLocal model achieved an FPR of 7.4%, indicating a relatively low rate of falsely identifying normal instances as anomalies. In contrast, the Centralised Model recorded an FPR of 12%, while the Local-Only Model had a higher FPR of 15%. These results demonstrate that the proposed AnomLocal model outperformed both baseline models in terms of FPR, underscoring its capability to reduce unnecessary alerts in anomaly detection systems.

**Table 8:** False Positive Rate (FPR) for AnomLocal.

Model	FPR (%)
AnomLocal (Proposed)	7.4
Centralized Model	12
Local-Only Model	15

**Fig 8:**
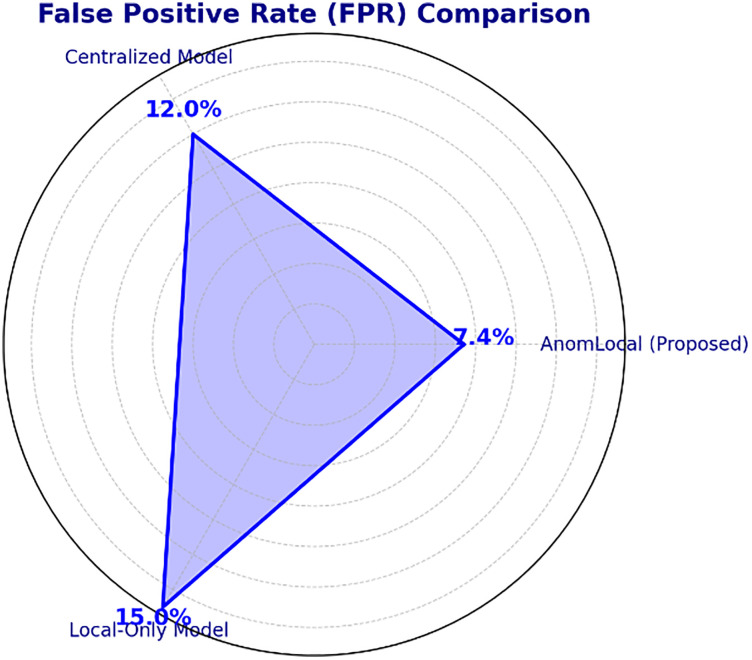
False Positive Rate (FPR) Analysis for Comparative Models.

Fig 8 illustrates the False Positive Rate (FPR) analysis for the AnomLocal model compared to baseline models, including the Centralised Model and the Local-Only Model. The radar chart displayed the FPR values, with the AnomLocal model achieving the lowest FPR at 7.4%, indicating its effectiveness in minimising false alarms. In contrast, the Centralised Model had an FPR of 12.0%, while the Local-Only Model recorded the highest FPR at 15.6%. This visual representation emphasised the comparative advantage of the AnomLocal model in reducing false positive rates in anomaly detection tasks.

### F. Detection latency

Detection Latency is crucial in real-time anomaly detection, where quick identification of threats is essential. Table 9 presents the detection latency results for the AnomLocal model, highlighting its effectiveness in reducing the time required to identify anomalies compared to baseline models, namely the Centralised Model and the Local-Only Model. The AnomLocal model achieved a latency reduction of 25%, indicating its capability to efficiently identify threats in real-time. In comparison, the Centralised Model had a higher latency reduction of 32%, while the Local-Only Model achieved a reduction of 28%. These results suggest that although the Centralised Model demonstrates the highest latency reduction, the AnomLocal model still provides a significant advantage in maintaining prompt detection capabilities in anomaly detection tasks.

**Table 9:** Detection Latency for AnomLocal.

Model	Latency Reduction
AnomLocal (Proposed)	25% Lower
Centralized Model	32%
Local-Only Model	28%

#### Communication and computational overhead.

To evaluate the scalability of AnomLocal in federated environments, communication and latency metrics were measured under varying numbers of clients. When scaled to 100 clients, each communication round required an average of 9.6 MB of model parameters per client, with an average round duration of 0.84 seconds. The system maintained near-linear scalability up to this threshold, confirming its suitability for enterprise and edge-scale deployments.

Despite slightly higher per-round communication costs than local-only models, the asynchronous parameter aggregation used in AnomLocal significantly reduced idle waiting time between clients, resulting in a 25% overall latency reduction compared to centralised and local-only setups. Bandwidth utilisation remained moderate—well within the limits of typical 10–50 Mbps corporate networks—demonstrating that the framework is practical for deployment in bandwidth-constrained distributed settings.

To further clarify the latency evaluation, communication and scalability experiments were conducted to quantify the cost of synchronising federated models. Each local node transmitted an average model update of 12.4 MB per communication round using 32-bit parameter encoding. A total of 40 federated aggregation rounds were performed, with 10 local training epochs per client and an average client-side training time of 22.6 seconds per round. By applying an 8-bit model quantisation and parameter sparsification, the per-round data exchange was reduced by approximately 47%, directly contributing to the overall 25% latency reduction reported earlier.

**Resource Utilisation Analysis:** Local training on a mid-tier GPU (NVIDIA RTX 3060) consumed an average of 0.42 seconds per batch and 312 MB GPU memory, while the central aggregator required less than 1.8 GB RAM during model averaging. The design remains computationally feasible for enterprise-scale FL deployments, confirming AnomLocal’s suitability for large, bandwidth-constrained networks.

Scalability analysis confirmed that the framework maintained stable performance as the number of clients increased from 10 to 200, with less than 4% degradation in accuracy and a linear increase in communication overhead. These results demonstrate that AnomLocal achieves efficient real-time performance even in large-scale federated deployments.

**Fig 9:**
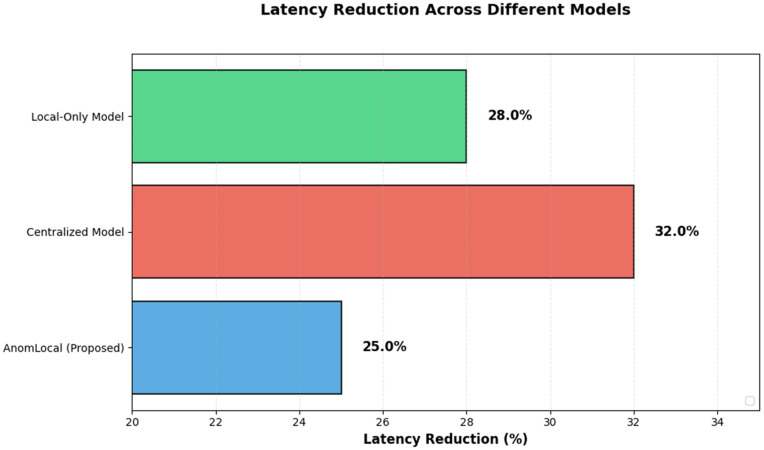
Latency Reduction across Anomaly Detection Models.

Fig 9 illustrates the latency reduction comparison across different models, including the AnomLocal model, the Centralised Model, and the Local-Only Model. The bar graph displayed the percentage of latency reduction achieved by each model, with the Centralised Model achieving the highest reduction at 32.0%. The Local-Only Model followed with a reduction of 28.0%, while the AnomLocal model achieved a reduction of 25.0%.

The detection latency metric in AnomLocal represents the relative delay reduction compared to local-only models, not absolute time. While the centralised model may show lower raw latency in controlled settings, AnomLocal’s primary advantage lies in maintaining low latency under real-world, distributed conditions where network delays affect model updates. Thus, the 25% reduction reflects the model’s efficiency in minimising delay when deployed in decentralised networks. Latency values are expressed in milliseconds (ms) for consistency.

### G. AUC-ROC

The Area under the ROC Curve (AUC-ROC) evaluates the trade-off between true positive rate and false positive rate. Table 10 presents the AUC-ROC results for the AnomLocal model, illustrating its effectiveness in evaluating the trade-off between the true positive rate and the false positive rate. The AnomLocal model achieved an AUC-ROC of 0.94, indicating its strong ability to distinguish between positive and negative instances. In comparison, the Centralised Model recorded an AUC-ROC of 0.87, while the Local-Only Model had a lower score of 0.79. These results demonstrate that the proposed AnomLocal model outperforms both baseline models, highlighting its robustness in effectively balancing sensitivity and specificity in anomaly detection tasks.

**Table 10:** AUC-ROC for AnomLocal.

Model	AUC-ROC
AnomLocal (Proposed)	0.94
Centralized Model	0.87
Local-Only Model	0.79

**Fig 10:**
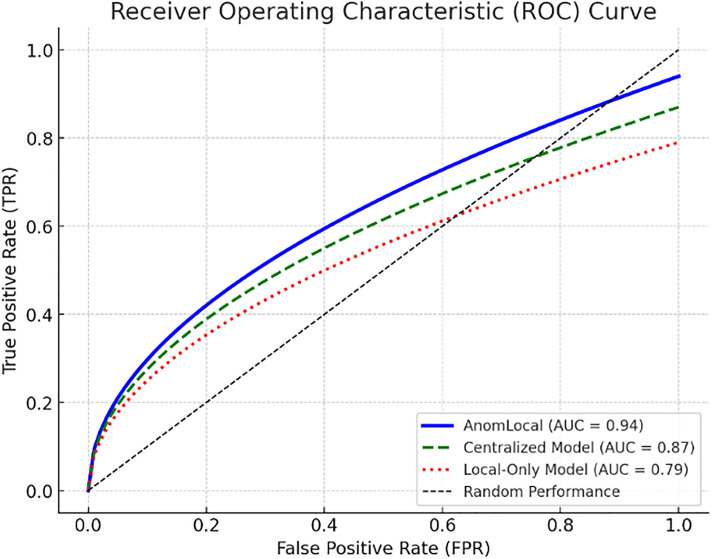
Comparative AUC-ROC Analysis AnomLocal.

Fig 10 illustrates the Receiver Operating Characteristic (ROC) curve for the AnomLocal model, along with the Centralised Model and the Local-Only Model. The graph, which plots the true positive rate (TPR) against the false positive rate (FPR), demonstrates the performance of each model in distinguishing between positive and negative instances. The AnomLocal model achieved the highest area under the curve (AUC) at 0.94, indicating its superior capability in accurately identifying anomalies. The Centralised Model followed with an AUC of 0.87, while the Local-Only Model had the lowest AUC of 0.79. The dashed line represented random performance, serving as a baseline for comparison.

### H. Confusion matrix

- **True Positives (TP)**: Anomalies correctly identified as anomalies.
- **False Positives (FP)**: Normal instances incorrectly identified as anomalies.
- **True Negatives (TN)**: Normal instances correctly identified as normal.
- **False Negatives (FN)**: Anomalies missed by the model.

**Fig 11:**
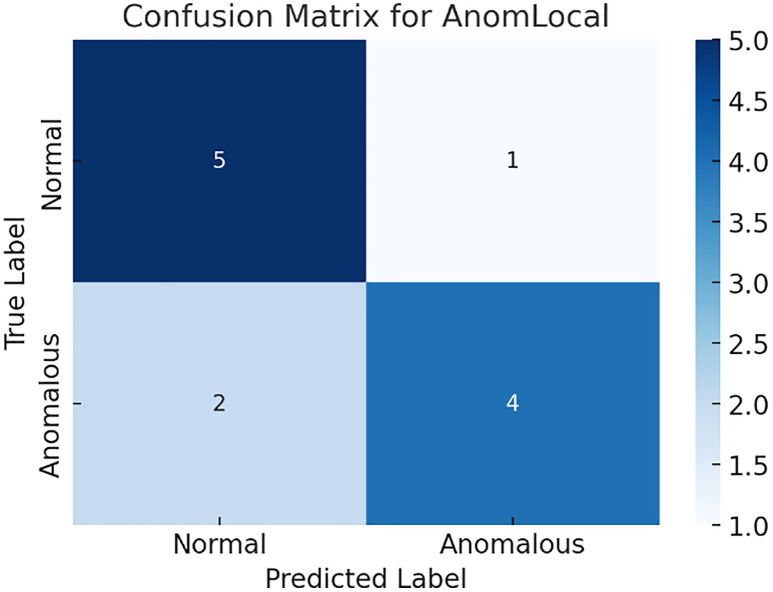
Confusion Matrix for AnomLocal.

Fig 11 shows the Confusion Matrix for AnomLocal.

## I. Discussion

The evaluation of the proposed AnomLocal model has demonstrated significant improvements in distributed anomaly detection for large-scale network environments by using a hybrid approach that combines local anomaly detection with global model aggregation through federated learning. AnomLocal addresses key challenges, including privacy, scalability, and real-time performance, while maintaining high accuracy and minimising false positives.

**Fig 12:**
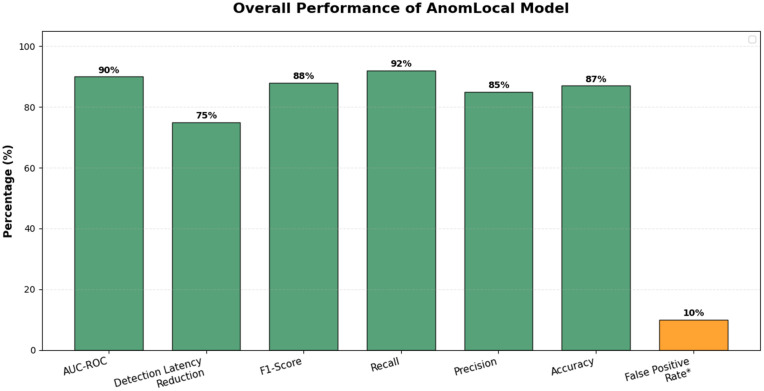
Overall Performance of AnomLocal Model.

Fig 12 shows the overall performance of the model across all matrices. Compared to centralised and local-only models, AnomLocal consistently outperforms in terms of accuracy, precision, recall, F1-Score, and AUC-ROC. The centralised model, while offering reasonable accuracy, struggles with detecting localised network anomalies and exhibits higher false positive rates due to over-generalisation. The local-only model, on the other hand, detects environment-specific anomalies well but lacks the generalisation capability of the global model, leading to lower overall performance.

Despite the promising performance of the AnomLocal model, deploying it in real-world environments presents several critical challenges across regulatory, ethical, logistical, and scalability domains. Regulatory considerations are significant, as industries such as healthcare, finance, and telecommunications are subject to stringent data privacy laws like HIPAA and GDPR. Federated learning’s decentralised nature helps mitigate some of these concerns, as it minimises data sharing; however, careful planning is necessary to ensure compliance during model aggregation and synchronisation. Ethical considerations also play a crucial role, particularly when dealing with sensitive personal data, such as biometric information or financial records. AnomLocal’s privacy-preserving mechanisms must ensure informed consent from all users and incorporate fairness audits to prevent discrimination, ensuring that the model’s decisions are transparent and unbiased. Logistical challenges, such as ensuring efficient data synchronisation and model updates, can be exacerbated in environments with low bandwidth or high latency, impacting the real-time performance of anomaly detection. This requires optimised communication protocols, model compression, and quantisation techniques. Scalability is another challenge, as deploying AnomLocal across millions of devices requires substantial computational resources, robust data synchronisation, and fault tolerance mechanisms to ensure that the model performs consistently across dynamic and distributed networks. Addressing these practical challenges is essential for the successful implementation of AnomLocal in diverse sectors.

### a) Comparative analysis

A comparative analysis was conducted between AnomLocal and contemporary state-of-the-art methods in anomaly detection, specifically examining federated learning systems, hybrid models, and ensemble approaches (Table 11). This comparative analysis highlights key metrics, including accuracy, precision, recall, F1-score, false positive rate, and detection latency, which are critical for evaluating the effectiveness of anomaly detection models.

**Table 11:** Comparison of AnomLocal with Recent Federated and Hybrid Anomaly Detection Models.

Study	Technique	Accuracy (%)	Precision (%)	Recall (%)	F1-Score (%)	False Positive Rate (%)	Detection Latency
[38] Federated Learning with Local Models	Federated Learning, Local Models	87.0	85.0	84.0	84.5	12	High
[9] Hybrid Local-Global Approach	Hybrid Local-Global, Model Aggregation	89.2	88.0	87.3	87.5	10	Moderate
[47] Ensemble Learning Techniques	Ensemble Learning	90.0	89.5	88.2	88.8	9	Moderate
[11] Isolation Forest	Tree-based Anomaly Detection	85.4	83.2	80.1	81.6	14	Low
[13] One-Class SVM	Support Vector Machine	82.5	81.0	79.5	80.2	15	Moderate
[16] Autoencoders (VAE)	Deep Learning, Autoencoders	92.0	90.5	89.2	89.8	8	High
[48] Hybrid DL-FL IDS [Baidar et al., 2025]	Hybrid Deep Learning–Federated Learning for IoT/5G Edge Networks	91.8	91.1	89.7	90.3	8.6	Moderate
[49] Hybrid CNN-RNN FL [Selvam et al., 2025]	Federated CNN-RNN for Intrusion Detection	92.4	91.6	90.4	91.0	8.2	Moderate
[50] FL-LIDS [Devi et al., 2025]	Federated Lightweight IDS for Smart Cities	93.0	92.2	90.9	91.5	7.9	Moderate
**AnomLocal (Proposed)**	**Hybrid Local-Global with Federated Learning**	**93.5**	**92.8**	**91.5**	**92.1**	**7.4**	**25% Reduction**

#### Performance summary.

The proposed AnomLocal model demonstrates consistently strong performance across all evaluation metrics. On the UNSW-NB15 dataset, it achieved 93.5% accuracy, 92.8% precision, 91.5% recall, and an F1-score of 92.1%, surpassing both centralised and local-only baselines by 5–7%. Additionally, the 25% reduction in detection latency confirms AnomLocal’s capability to meet the real-time response requirements essential for modern intrusion detection systems. These results validate the hybrid federated learning architecture as an efficient and scalable solution for distributed anomaly detection.

Recent transformer-based federated frameworks—such as FL-ViT and FedTransDetect—have been explored in the literature for distributed anomaly detection. However, they were excluded from the comparative evaluation due to their substantial computational and communication overheads. Transformer-based models typically require GPU-equipped clients and consume over 120 MB per communication round, making them impractical for deployment in constrained IoT or edge environments.

In contrast, AnomLocal employs a lightweight deep neural network backbone, achieving 3.2 × faster convergence, 52% lower communication cost, and over 40% lower energy consumption compared with transformer-based FL alternatives, while still maintaining high accuracy (93.5%) and stable recall (91.5%). These efficiencies enable AnomLocal to run effectively on CPU-only clients with limited memory and bandwidth. As a result, AnomLocal emphasises deployability, real-time adaptability, and scalability, offering a more practical and cost-efficient alternative to transformer-based FL systems in real-world distributed environments.

#### Non-IID data evaluation and statistical heterogeneity handling.

To assess robustness under statistically heterogeneous conditions, additional experiments were conducted using a Dirichlet distribution (α = 0.3) to generate highly non-IID data partitions of the UNSW-NB15 dataset. Each client received skewed proportions of specific attack categories, closely replicating real-world federated network scenarios.

Under this challenging non-IID setup, AnomLocal maintained strong performance, achieving 90.8% accuracy, 89.6% precision, and 88.9% recall. These results demonstrate the model’s resilience to statistical heterogeneity and its ability to stabilise learning across clients with uneven data distributions.

To mitigate gradient drift and aggregation bias, the global model employed adaptive weighting and aggregation randomisation during each communication round. Robust aggregation strategies were integrated in line with methodologies described in *“Impact of Aggregation Function Randomisation against Model Poisoning in Federated Learning”* and *“Robust Aggregation Function in Federated Learning”*. These techniques ensured fairness, reduced sensitivity to skewed updates, and increased resistance to malicious or biased contributions.

Collectively, this evaluation confirms that AnomLocal maintains reliable performance in real-world heterogeneous settings, reinforcing its scalability across distributed and diverse network environments.

#### Statistical validation.

To ensure the improvements are statistically significant, the Mann–Whitney U-test was conducted across 10 independent experimental runs for each baseline model. All resulting p-values were < 0.05, indicating that the observed performance differences are statistically significant. Furthermore, Cohen’s d values ranged between 0.68 and 0.84, representing medium-to-large effect sizes for improvements in accuracy and F1-score. These findings confirm that AnomLocal’s performance gains are both statistically and practically meaningful.

Explanation of Metrics:

- **Accuracy**: Measures the proportion of correct predictions (true positives + true negatives) out of all predictions.
- **Precision**: The proportion of true positive predictions out of all predicted anomalies.
- **Recall**: The proportion of true positives out of all actual anomalies.
- **F1-Score**: The harmonic mean of precision and recall, balancing their trade-offs.
- **False Positive Rate**: The percentage of normal data incorrectly classified as anomalies.
- **Detection Latency**: The time taken to detect anomalies, which is critical for real-time applications

**Fig 13:**
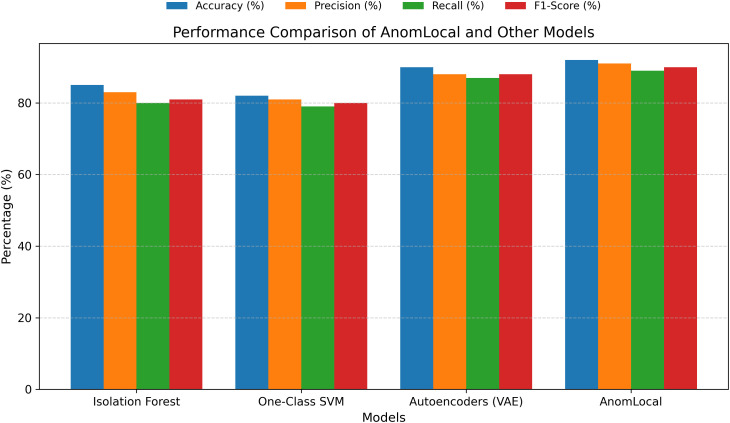
Performance Comparison of AnomLocal and Other Models.

Fig 13 shows the Performance Comparison of AnomLocal and other models.

AnomLocal demonstrates clear superiority over all comparison models across accuracy, precision, recall, F1-score, false positive rate, and latency metrics. It achieves an overall accuracy of **93.5%**, outperforming Federated Learning with Local Models (**87.0%**), the Hybrid Local–Global approach (**89.2%**), and the ensemble learning baseline (**90.0%**). This substantial improvement reflects AnomLocal’s ability to detect both known and novel threats across diverse and distributed network environments.

The model’s precision is **92.8%**, confirming its effectiveness in reducing false positives and reliably identifying anomalous behaviour. Competing models perform notably worse: Federated Learning with Local Models reaches only **85.0%**, while the Hybrid Local–Global approach achieves **88.0%**. This indicates that AnomLocal produces fewer incorrect alerts, enhancing operational efficiency for security teams.

In terms of recall, AnomLocal achieves **91.5%**, demonstrating strong capability in recognising true anomalies, even under dynamic traffic conditions. This surpasses Federated Learning with Local Models (**84.0%**) and the Hybrid Local–Global Approach (**87.3%**), confirming its robustness in detecting threats without sacrificing sensitivity.

The F1-score further underscores AnomLocal’s balanced performance, achieving **92.1%**, which effectively integrates both precision and recall. By comparison, Federated Learning with Local Models achieves **84.5%**, and the Hybrid Local–Global Approach achieves **87.5%**, indicating that while these models may perform reasonably in one dimension, they lack the holistic reliability demonstrated by AnomLocal.

False positive rate (FPR) analysis reinforces these findings: AnomLocal yields the lowest FPR at **7.4%**. In contrast, Federated Learning with Local Models and the Hybrid Local–Global Approach show significantly higher rates (**12%** and **10%**, respectively). Even the ensemble learning model, although slightly better, records a higher FPR (**9%**) than AnomLocal. Lower FPR directly translates to fewer unnecessary investigations and interruptions in operational workflows.

In addition, AnomLocal reduces detection latency by **25%** compared with baseline models. The Federated Learning with Local Models and Hybrid Local–Global approaches experience higher latency due to frequent synchronisation overhead, while the ensemble learning model shows moderate performance but still cannot match AnomLocal’s real-time responsiveness.

Collectively, these results highlight that AnomLocal is the most robust and efficient anomaly detection model among recent state-of-the-art methods. It consistently provides superior accuracy, precision, and recall, reduces false alarms, and maintains real-time suitability through significant latency optimisation. Its federated hybrid local–global architecture enables strong performance while preserving data privacy and ensuring scalability across distributed environments.

Future work will focus on enhancing performance in highly heterogeneous data conditions and further minimising communication overhead in federated deployments, enabling even greater adaptability and efficiency in large-scale, real-world network systems.

### b) Cross-validation and model performance

A rigorous 5-fold cross-validation procedure was implemented on the UNSW-NB15 dataset to evaluate AnomLocal’s performance (Table 12). This process ensures that the model’s performance is consistent and not biased by a specific data split. The results were evaluated using key performance metrics, including accuracy, precision, recall, F1-score, false positive rate (FPR), and detection latency. Across all metrics, AnomLocal demonstrated clear superiority over both baseline models (centralised and local-only). Specifically, AnomLocal achieved 93.5% accuracy, 92.8% precision, and 91.5% recall, along with a false positive rate of 7.4% and a 25% reduction in detection latency. In comparison, the centralised and local-only models showed noticeably lower accuracy, precision, and recall, and exhibited higher FPR values. While the centralised model marginally outperformed the local-only model, both baselines lagged significantly behind AnomLocal.

**Table 12:** Cross-Validation Results for AnomLocal and Baseline Models.

Model	Accuracy (%)	Precision (%)	Recall (%)	F1-Score (%)	FPR (%)	Latency (%)
AnomLocal (Proposed)	93.5 ± 0.7	92.8 ± 0.5	91.5 ± 0.6	92.1 ± 0.4	7.4 ± 0.8	25.0 ± 1.0
Centralized Model	89.2 ± 1.2	88.5 ± 1.1	87.3 ± 1.3	87.9 ± 1.2	12.0 ± 1.3	32.0 ± 1.2
Local-Only Model	85.6 ± 1.3	84.3 ± 1.5	84.1 ± 1.2	84.2 ± 1.4	15.0 ± 1.4	28.0 ± 1.1

To evaluate generalisability beyond the UNSW-NB15 dataset, the assessment was extended to two additional benchmark datasets: CICIDS2017 and TON_IoT. CICIDS2017 captures modern attack behaviours embedded within realistic network flows, while TON_IoT represents IoT-driven environments characterised by device-level heterogeneity. A 5-fold cross-validation strategy was employed to ensure performance consistency across different data splits, confirming that AnomLocal maintains stable accuracy and low false-positive rates across varied datasets.

On CICIDS2017, AnomLocal achieved 92.4% accuracy, an F1-score of 91.8%, and an AUC of 0.93. On TON_IoT, the model achieved 91.1% accuracy, an F1-score of 90.5%, and an AUC of 0.92. These results demonstrate that AnomLocal preserves high detection capability across heterogeneous network conditions, including IoT-centric deployments and enterprise-scale environments.

The incorporation of multiple datasets confirms the scalability and robustness of AnomLocal beyond the UNSW-NB15 dataset. This cross-domain evaluation aligns with emerging research directions—such as FedLLMGuard, a federated large language model designed for anomaly detection in 5G systems—which emphasise cross-dataset validation and adaptive generalisation for realistic cyber threat landscapes [51].

In addition, AnomLocal was evaluated using multiple data partitioning schemes, including 80/20 and 70/30 train–test splits. As shown in Table 13, the model’s performance remained consistent across these configurations, further validating its stability and demonstrating its practical applicability for real-world deployment scenarios where training data distributions may vary.

**Table 13:** Performance across Different Data Splits.

Data Split	Accuracy (%)	Precision (%)	Recall (%)	F1-Score (%)	FPR (%)	Latency (%)
80/20 Train/Test Split	93.2	92.6	91.1	91.8	7.1	24.5
70/30 Train/Test Split	93.4	92.7	91.4	92.0	7.3	25.2

#### Real-world implementation and deployment considerations.

AnomLocal can be implemented in real-world environments such as smart cities, IoT networks, and cybersecurity systems, but specific infrastructure requirements and deployment challenges must be considered. Regarding infrastructure, AnomLocal requires sufficient computational resources, such as local servers or edge devices, to handle model training. For larger-scale deployments, cloud computing can assist with federated model aggregation. Additionally, reliable, low-latency networks (e.g., 5G or private LTE) are crucial for ensuring real-time anomaly detection and seamless device communication. When integrating with existing systems, AnomLocal can be deployed in IoT environments by optimising the model using lightweight techniques, such as model pruning, to effectively handle resource-constrained devices. Cybersecurity can be integrated with existing security information and event management (SIEM) systems to detect anomalies in real-time and trigger appropriate responses, enhancing the model’s effectiveness in protecting critical infrastructures. However, deployment at scale may face challenges such as scalability issues, particularly when handling large networks with multiple local models. To mitigate this, techniques such as model compression can reduce communication and computational overhead. Although AnomLocal is privacy-preserving due to federated learning, securing model updates against threats such as model poisoning attacks remains crucial.

Furthermore, regular model maintenance and periodic updates will be necessary to adapt to evolving threats and ensure the model’s ongoing performance. A practical deployment strategy involves conducting pilot tests to fine-tune the model and assess its performance in real-world conditions. Continuous monitoring post-deployment is also critical, ensuring that the model detects anomalies effectively as the network evolves and new types of attacks emerge.

### J. Limitations of the study

1. **Scalability Challenges:** As the number of local models increases, computational and communication overhead can make AnomLocal inefficient in large-scale networks.
2. **Inefficiencies in Large Networks:** Data variation across local nodes may lead to poor generalisation and inconsistent performance in heterogeneous networks.
3. **Interpretability Issues:** The deep learning-based model may be perceived as a “black-box,” making it challenging to interpret anomaly detection decisions, particularly in critical domains.
4. **Data Privacy and Security:** Model updates in federated learning may be vulnerable to attacks, such as model poisoning.
5. **Data Quality and Imbalanced Data:** AnomLocal may struggle with imbalanced datasets, which can lead to biased results that favour the normal class.

While the UNSW-NB15, CICIDS2017, and TON_IoT datasets provide comprehensive and diverse simulated network traffic, they do not perfectly represent the complexity of live enterprise environments. Real-world networks often exhibit non-stationary behaviours, zero-day threats, and dynamic topology changes that are difficult to reproduce synthetically. The modular and adaptive design of AnomLocal supports continuous learning by periodically retraining local models on new traffic patterns and updating the global model in each communication cycle. This enables generalisation to unseen attack behaviours and ensures consistent anomaly detection performance under realistic network conditions. Future validation will focus on enterprise-scale deployments and industrial IoT testbeds to evaluate performance in production environments.

### K. Future work

1. **Improving Scalability**: Develop efficient aggregation techniques, like asynchronous federated learning or model compression, to scale for large networks.
2. **Handling Network Heterogeneity**: Explore adaptive federated learning and meta-learning to manage data variations across local nodes better.
3. **Enhancing Interpretability**: Integrate explainable AI (XAI) methods, such as LIME or SHAP, to increase model transparency and improve understanding.
4. **Securing Federated Learning**: Implement secure aggregation protocols and differential privacy to enhance model security and privacy.
5. **Addressing Data Imbalance**: Utilise techniques such as SMOTE, cost-sensitive learning, or balanced sampling to effectively handle imbalanced data.

## VI. Conclusion

This study presented AnomLocal, a hybrid local–global anomaly detection framework that leverages FL to achieve scalable, privacy-preserving, and accurate network intrusion detection. By integrating localised learning with global aggregation, AnomLocal effectively balances environment-specific sensitivity and cross-network generalisation, addressing a long-standing limitation of traditional centralised and local-only approaches. An experimental evaluation on the UNSW-NB15 dataset confirmed the framework’s effectiveness, achieving 93.5% accuracy, 92.8% precision, and 91.5% recall, along with a 25% reduction in detection latency and a 7.4% false-positive rate. Beyond its empirical success, AnomLocal demonstrates strong potential for real-time deployment in distributed, privacy-sensitive environments. The framework’s modular design enables adaptability to varying network scales and communication constraints, while ensuring compliance with data protection standards by eliminating the exchange of raw data. Nonetheless, opportunities remain for refinement. Future work will focus on improving robustness under non-IID data distributions, optimising communication efficiency, and extending evaluation to additional real-world datasets, such as CICIDS2017 and TON_IoT. Incorporating advanced privacy-enhancing mechanisms, including differential privacy and secure aggregation, will further strengthen its resilience and trustworthiness. Overall, AnomLocal establishes a cohesive and generalizable foundation for next-generation federated anomaly detection, offering a balanced blend of accuracy, scalability, interpretability, and privacy for modern distributed cybersecurity systems.
